# Antioxidant Properties and Cytoprotective Effect of *Pistacia lentiscus* L. Seed Oil against 7β-Hydroxycholesterol-Induced Toxicity in C2C12 Myoblasts: Reduction in Oxidative Stress, Mitochondrial and Peroxisomal Dysfunctions and Attenuation of Cell Death

**DOI:** 10.3390/antiox10111772

**Published:** 2021-11-05

**Authors:** Imen Ghzaiel, Amira Zarrouk, Thomas Nury, Michela Libergoli, Francesca Florio, Souha Hammouda, Franck Ménétrier, Laure Avoscan, Aline Yammine, Mohammad Samadi, Norbert Latruffe, Stefano Biressi, Débora Levy, Sérgio Paulo Bydlowski, Sonia Hammami, Anne Vejux, Mohamed Hammami, Gérard Lizard

**Affiliations:** 1 Team ‘Biochemistry of the Peroxisome, Inflammation and Lipid Metabolism’ EA7270/Inserm, University Bourgogne Franche-Comté, 21000 Dijon, France; imenghzaiel93@gmail.com (I.G.); thomas.nury@u-bourgogne.fr (T.N.); alineyammine5@gmail.com (A.Y.); norbert.latruffe@u-bourgogne.fr (N.L.); anne.vejux@u-bourgogne.fr (A.V.); 2 Lab-NAFS ‘Nutrition—Functional Food & Vascular Health’, Faculty of Medicine, University of Monastir, LR12ES05, Monastir 5000, Tunisia; souhahammouda51@gmail.com (S.H.); sonia.hammami@fmm.rnu.tn (S.H.); mohamed.hammami@fmm.rnu.tn (M.H.); 3 Faculty of Sciences of Tunis, University Tunis-El Manar, Tunis 2092, Tunisia; 4 Faculty of Medicine, University of Sousse, Sousse 4000, Tunisia; 5 Department of Cellular, Computational and Integrative Biology (CIBio) and Dulbecco Telethon Institute, University of Trento, 38123 Trento, Italy; michela.libergoli@unitn.it (M.L.); francesca.florio@unitn.it (F.F.); stefano.biressi@unitn.it (S.B.); 6 Centre des Sciences du Goût et de l’Alimentation, AgroSup Dijon, CNRS, INRAE, Université Bourgogne Franche-Comté, 21065 Dijon, France; franck.menetrier@inrae.fr; 7 Agroécologie, AgroSup Dijon, CNRS, INRAE, University Bourgogne Franche-Comté, Plateforme DimaCell, 21000 Dijon, France; laure.avoscan@inrae.fr; 8 LCPMC-A2, ICPM, Department of Chemistry, University Lorraine, Metz Technopôle, 57070 Metz, France; mohammad.samadi@univ-lorraine.fr; 9 Lipids, Oxidation and Cell Biology Team, Laboratory of Immunology (LIM19), Heart Institute (InCor), Faculdade de Medicina, Universidade de São Paulo, São Paulo 05403-900, Brazil; d.levy@hc.fm.usp.br (D.L.); spbydlow@usp.br (S.P.B.); 10 National Institute of Science and Technology in Regenerative Medicine (INCT-Regenera), CNPq, Rio de Janeiro 21941-902, Brazil

**Keywords:** aging, 7β-hydroxycholesterol, mitochondria, C2C12 myoblasts, oxidative stress, peroxisome, *Pistacia lentiscus* L. seed oil, sarcopenia

## Abstract

Aging is characterized by a progressive increase in oxidative stress, which favors lipid peroxidation and the formation of cholesterol oxide derivatives, including 7β-hydroxycholesterol (7β-OHC). This oxysterol, which is known to trigger oxidative stress, inflammation, and cell death, could contribute to the aging process and age-related diseases, such as sarcopenia. Identifying molecules or mixtures of molecules preventing the toxicity of 7β-OHC is therefore an important issue. This study consists of determining the chemical composition of Tunisian *Pistacia lentiscus* L. seed oil (PLSO) used in the Tunisian diet and evaluating its ability to counteract the cytotoxic effects induced by 7β-OHC in murine C2C12 myoblasts. The effects of 7β-OHC (50 µM; 24 h), associated or not with PLSO, were studied on cell viability, oxidative stress, and on mitochondrial and peroxisomal damages induction. α-Tocopherol (400 µM) was used as the positive control for cytoprotection. Our data show that PLSO is rich in bioactive compounds; it contains polyunsaturated fatty acids, and several nutrients with antioxidant properties: phytosterols, α-tocopherol, carotenoids, flavonoids, and phenolic compounds. When associated with PLSO (100 µg/mL), the 7β-OHC-induced cytotoxic effects were strongly attenuated. The cytoprotection was in the range of those observed with α-tocopherol. This cytoprotective effect was characterized by prevention of cell death and organelle dysfunction (restoration of cell adhesion, cell viability, and plasma membrane integrity; prevention of mitochondrial and peroxisomal damage) and attenuation of oxidative stress (reduction in reactive oxygen species overproduction in whole cells and at the mitochondrial level; decrease in lipid and protein oxidation products formation; and normalization of antioxidant enzyme activities: glutathione peroxidase (GPx) and superoxide dismutase (SOD)). These results provide evidence that PLSO has similar antioxidant properties than α-tocopherol used at high concentration and contains a mixture of molecules capable to attenuate 7β-OHC-induced cytotoxic effects in C2C12 myoblasts. These data reinforce the interest in edible oils associated with the Mediterranean diet, such as PLSO, in the prevention of age-related diseases, such as sarcopenia.

## 1. Introduction

Aging is characterized by a progressive loss of physiological functions, coupled with a reduction in the ability to maintain homeostasis [1]. One of the hallmark effects of aging is sarcopenia, which is widely defined as an age-related progressive decline in skeletal muscle mass, strength, and function [2,3]. From the age of 30, humans lose approximately 3–8% of muscle mass per decade with an accelerated rate of decline after the age of 60 [4,5,6]. This decline leads to limited functional mobility in older adults [7], but it may also aggravate their vulnerability [8]. Indeed, skeletal muscle is the largest organ in the human body. It accounts for 30–40% of total body weight [9,10] and plays a primordial role in locomotion [11], respiration [12], thermogenesis [13], and regulation of lipids (fatty acids, cholesterol) [14,15] and glucose metabolism [16]. The aging skeletal muscle is marked by the development of an alteration of energy substrates use. In fact, in response to insulin stimulation, mitochondria of elderly subjects are unable to move from lipid oxidation to carbohydrate oxidation as do mitochondria of young subjects, showing a loss of metabolic flexibility during aging [17]. These data suggest an alteration in metabolic dynamism and highlight the inability of muscle cells to adapt to environmental variations. It is therefore important to better understand the underlying mechanisms leading to sarcopenia but also to identify the chemical epigenetic factors that may promote it. In addition, during aging, to allow the maintenance of muscle mass, strength and quality, and to act on these parameters, it is important to identify natural or synthetic molecules, as well as mixtures of molecules that can be provided in the form of food supplements, or functional foods [18]. An adapted diet, as well as foods acting on the muscle, must also be considered. Several aging mechanisms have been identified, including telomere shortening, genomic instability, epigenetic alterations, and organelle dysfunction (mainly mitochondrial changes), which can trigger cellular senescence [18]. In addition, among the parameters that can affect the aging of skeletal muscle, oxidative stress is one of the major contributors that can favor skeletal muscle damage [19,20]. Skeletal muscle consumes large quantities of oxygen compared to other tissues, resulting in higher amounts of reactive oxygen species (ROS) [21]. This increase in ROS levels could be due to two main factors: (i) altered functions of the mitochondrial respiratory chain; and (ii) impairment in cellular antioxidant defense mechanisms [22]. These ROS contribute to increased uptake of both glucose and cholesterol into the cells [23]. Cholesterol is a major component of cellular membranes, including the sarcolemma of skeletal muscle [24]. In the presence of oxidative stress, glucose and cholesterol can undergo auto-oxidation by a free-radical mechanism. The cholesterol oxide derivatives (oxysterols) formed by cholesterol auto-oxidation in oxidative stress conditions mainly correspond to those that are oxidized at position C7 (7-ketocholesterol (7KC) and 7β-hydroxycholesterol (7β-OHC)) [25,26,27,28]. Interestingly, 7KC and 7β-OHC have similar cytotoxic effects (although the induction of cell death is faster with 7β-OHC) and these two oxysterols can be interconverted: the enzyme 11β-hydroxysteroid dehydrogenase-1 (11β-HSD1) converts 7KC into 7β-OHC, whereas 11β-hydroxysteroid dehydrogenase-2 (11β-HSD2) converts 7β-OHC into 7KC [29,30]. In addition, these oxysterols have been identified as key elements in the development of age-related diseases: cardiovascular diseases, neurodegenerative diseases (Alzheimer’s and Parkinson’s), and ocular diseases (cataract, age-related macular degeneration) [28,31,32,33]. On various cell lines from different species, 7KC and 7β-OHC but also 24S-hydroxycholesterol trigger a mode of cell death by oxiapoptophagy, which includes oxidative stress and mitochondrial, lysosomal, and peroxisomal dysfunction, leading to an apoptotic mode of cell death associated with autophagic criteria [30,34,35,36]. Oxysterols are also involved in many physiologic processes: regulation of cholesterol metabolism [37] and RedOx homeostasis [38]; control of inflammation, including cytokine production [39]; albumin synthesis [40]; and cell differentiation [41]. Despite the considerable interest in oxysterols in the aging process, the impact of these molecules on skeletal muscle cells has not yet been studied. On human promonocytic U937 cells, 7KC, 7β-OHC and 5β,6β-epoxicholesterol showed high cytotoxicity, characterized by a high percentage of dead cells, overproduction of ROS, and secretion of pro-inflammatory cytokines [42]. It has been shown that the most toxic oxysterol on U937 cells, but also on other cells, was 7β-OHC [30,42].

Therefore, to mimic an age-related pro-oxidant environment, C2C12 murine myoblasts cultured in the presence of 7β-OHC were used as a cellular model. The effects of 7β-OHC were characterized on C2C12 and cytoprotective agents were investigated. The effect of α-tocopherol that protects many cells from the toxicity induced by 7β-OHC and 7KC was also analyzed [28] as well as that of *Pistacia lentiscus* L. seed oil (PLSO), widely used in the Tunisian diet. It is well known that vegetable oils are a valuable source of natural antioxidants, playing a crucial role in the improvement of human health [43] and in delaying muscle atrophy [44]. One of the most well-known powerful medicinal plants is *Pistacia lentiscus* L. (PL). PL is one of the Mediterranean’s most valuable and important aromatic bushes. Its application in traditional medicine has increased over time and it is used as a therapeutic agent in the treatment of scabies, rheumatism, the manufacture of anti-diarrheal medicine, and in minor burns [45,46,47]. Extracts of different parts of the plant show various activities, such as antioxidant, anti-inflammatory, anti-proliferative, and neuroprotective effects [48,49,50,51]. The fruits of PL give an edible oil with high nutritional value due to its richness in unsaturated fatty acids, such as oleic acid (C18:1 n−9) and linoleic acid (C18:2 n−6) [52]. PLSO also has a comparable carotenoid and total polyphenols content than olive oil [53,54], and it has been demonstrated that it has antioxidant and protective effects against various diseases associated with oxidative stress [55,56].

In the present study, the first objective was to determine the biochemical composition of PLSO in polyphenols, flavonoids, β-carotene, fatty acids, phytosterols, and α-tocopherol, and its antioxidant properties. Furthermore, among the sarcopenic patients studied, as the level of 7β-OHC (mainly formed by cholesterol auto-oxidation, considered as a marker of oxidative stress, and known for its important pro-oxidant properties) was significantly higher than in non-sarcopenic subjects, the second objective of the study was to evaluate the ability of PLSO, compared to α-tocopherol, to attenuate the cytotoxic effects induced by 7β-OHC on murine C2C12 myoblasts; specifically, the effects on cell proliferation and viability, plasma membrane integrity, oxidative stress, and mitochondrial and peroxisomal status.

## 2. Material and Methods

### 2.1. Chemical Profile of Pistacia lentiscus *L*. Seed Oil

#### 2.1.1. Seed Material and Oil Extraction

The mature fruits of PL (lentisk-mastic tree) were collected in November 2019 from plants growing in the region of Tabarka (extreme north-west of Tunisia). After the harvest, fruits were ground using an ordinary grinder; the resulting paste was then manually mixed and let to stand overnight in a refrigerator. The next day, the paste was macerated in cold water. Subsequently, the mixture was placed in a water bath to prevent direct exposure of the ground material to the heat and thus degradation of the oil quality. After filtration, the oil was separated from the water by a decantation process. The procedure of *Pistacia lentiscus* L. seed oil is summarized in Supplementary Figure S1. The oils obtained were stored and maintained at 4 °C for further analyses.

#### 2.1.2. Colorimetric Determination of Total Phenolics, Flavonoids, and Carotenoids Contents of *Pistacia lentiscus L*. Seed Oil

The quantification of total phenolics and flavonoids was preceded by an extraction performed as follows: 4 g of PLSO was mixed thoroughly with 2 mL of n-hexane followed by the addition of 4 mL of methanol/water (60:40, *v/v*). The mixture was vortexed vigorously and centrifuged at 1490× *g* for 3 min to separate the two phases. The hydroalcoholic phase was collected, and the hexanic phase was re-extracted two more times with 4 mL of methanol/water (60:40, *v/v*) solution. Finally, the hydroalcoholic fraction obtained was combined, washed with 4 mL of n-hexane, and stored at −20 °C until analysis.

Phenolic compounds: Total phenolic compounds content was assayed by the Folin–Ciocalteau’s method [57]. Briefly, 200 μL of the combined hydroalcoholic fraction or standard gallic acid solutions was mixed thoroughly with 1 mL of freshly prepared Folin–Ciocalteau reagent and 0.8 mL of 7.5% sodium carbonate (Na_2_CO_3_). After incubation for 30 min in the dark at room temperature, the absorbance was measured at 765 nm and the results were expressed as mg of gallic acid equivalent per g of sample.

Flavonoids: Quantification of total flavonoids was determined using an aluminum chloride (AlCl_3_) colorimetric assay [58]. A volume of 100 μL of the combined hydroalcoholic fraction or standard catechin solutions was combined with 400 μL of distilled water and subsequently with 30 μL of 5% sodium nitrite (NaNO_2_) solution. After 5 min, 20 μL of a 10% AlCl_3_ solution and 200 μL of 1 M Na_2_CO_3_ solution were added. The final volume was adjusted with distilled water and mixed thoroughly. The absorbance was recorded at 510 nm, and the concentrations were expressed as mg of catechin equivalent per g of the sample.

Carotenoids: Total carotenoids content was measured by a colorimetric assay according to the method previously described by Dhibi et al. [59], and was expressed using the following formula:

Carotenoids = 𝐴max × (10^5^/2.65)

where 𝐴max is the maximum of absorption between 440 and 480 nm.

#### 2.1.3. Polyphenols Analysis

In order to identify and quantify the polyphenolic compounds in PLSO, we analyzed them by high-performance liquid chromatography (HPLC). To this end, 1 g of oil was dissolved in 6 mL of petroleum ether and was then purified on a silica SPE cartridge (previously conditioned by 6 mL petroleum ether 40–60). The cartridge was then washed with 12 mL of petroleum ether and dried under nitrogen for 10 min. Polyphenolic compounds were eluted with 8 mL of a mixture of methanol/distilled water 80/20 (*v/v*), and then with 8 mL of acetonitrile. The eluate was evaporated to dryness under reduced pressure at 50 °C. The residue was taken up in 400 µL of methanol. The resulting extract was filtered through a 0.45 µm nylon membrane. Polyphenol analysis was performed by HPLC on a Perkin Elmer series 200 apparatus equipped with an automatic injector, a quaternary pump, a column oven to Peltier effect, and a DAD detector. HPLC analyses were carried out using RPHPLC with a licrospher 100 RP-18 column (150 × 4.6 mm internal diameter (i.d.), 5 µm particle size, Merck). A gradient elution was programmed using as a mobile phase A, distilled water with an adjusted pH of 2.2 using trifluoroacetic acid (TFA), and as a mobile phase B, acetonitrile. The samples were eluted according to the following gradient: 0 to 5 min with solvent A 100%, and 5 to 50 min with solvent A 100 to 45%. The flow rate was set at 1 mL/min throughout the gradient and the column temperature was maintained at 25 °C. The injection volume was 20 µL, and UV detection was carried out at a wavelength of 280 nm. The calibration curve was constructed using quercetin standard solution at different concentration levels (25 to 400 mg quercetin/L). Data were expressed in mg equivalents quercetin/100 g of oil. Polyphenols analysis was realized by the Lara-Spiral laboratory (Couternon, France). The available polyphenol spectra present in the database of the Lara-Spiral company are listed in Supplementary Table S1.

#### 2.1.4. Fatty Acids Analysis

Total lipids were extracted from PLSO according to the method described by Moilanen and Nikkari [60]. C19:0 was used as the internal standard in the experiments realized in LR12ES05, Lab-NAFS ‘Nutrition—Functional Food & Vascular Health’, Monastir, Tunisia. Lipids were trans-methylated with 14% boron trifluoride in methanol (BF_3_-MeOH) using the method of Morrison and Smith [61]. Subsequently, fatty acid methyl esters were analyzed by gas chromatography (GC) under the same conditions described by Zarrouk et al. [62]. Fatty acids were identified by comparison with synthetic standards. This experiment was performed in triplicate and the data expressed either as g/100 g of oil (C19:0 used as internal standard) or as percentages of total fatty acids.

#### 2.1.5. Phytosterols Analysis

The analysis of phytosterols was realized as described by Zarrouk et al. [62]. Briefly, the quantification of phytosterols was based on an isolation of the unsaponifiable fraction and a silylation of the unsaponifiable fraction before direct injection in gas chromatography (GC). Then, GC separations were performed with a Hewlett-Packard (HP 5890D, Palo Alto, CA, USA) using a capillary column (30 m length × 0.25 mm i.d. film thickness 0.25 μm). Working conditions were as follow: carrier gas, helium; flow through the column, 1 mL/min; injector temperature, 290 °C; detector temperature, 290 °C; oven temperature, 260 °C; injection volume 1 μL. The phytosterols were characterized and quantified by gas chromatography–flame ionization detection (GC-FID). The spectra were compared with those of the internal library INRAE (Dijon, France). Likewise, they were confirmed with the NIST Mass Spectral Library and with the literature. The concentration of each PLSO sterol was expressed in mg/100 g of oil and obtained by three independent analyses.

#### 2.1.6. α-Tocopherol Analysis

PLSO was diluted 10 folds (*w/w*) with hexane. An amount of 5 mg diluted solution was mixed for 1 min with 200 µL of saline solution, 200 µL of ethanol/butylated hydroxy toluene (BHT) (Sigma-Aldrich; 50 mg/L) containing Tocol used as internal standard (1 ng/µL), and 500 µL of hexane. The extract was centrifuged at 10,000× *g* for 5 min at 4 °C. The upper layer (100 µL) was collected into a new tube and evaporated to dryness with a nitrogen stream. The dried extract was suspended with 50 μL of methanol/BHT (50 mg/L) and further centrifuged at 10,000× *g* for 5 min at 4 °C. The supernatant (40 µL) was finally transferred to an injection vial. Extract (2 µL) was injected with an 1100 autosampler into a Poroshell 120 EC-C18 (3 × 50 mm, 2.7 µm) maintained at 35 °C. Separation was achieved with a 1260 HPLC pump (Agilent Technologies, Santa Clara, CA, USA) using a linear gradient of methanol (90% up to 100% in 5 min, and maintained at 100% for 3 min). Detection was realized with a Fluorescence Light Detector (Agilent Technologies, Craven Arms, England) at λ _Exmax_ = 292 nm and λ _Emmax_ = 325 nm. Authentic α-tocopherol standards (0, 50, 100, 200, 400, 600, and 800 ng) were extracted with the same protocol as the PLSO sample. Area ratios of α-tocopherol (room temperature (RT) = 5.4 min) to tocol (RT = 4.0 min) were calculated for PLSO and calibrations standards. A linear calibration curve was used for the calculations. The α-tocopherol analysis was realized by the lipidomic analytical platform (LAP, Dijon, France).

### 2.2. Antioxidant Activity of Pistacia lentiscus *L*. Seed Oil

The hydroalcoholic fraction of PLSO was also used for the quantification of antioxidants activities with DPPH, FRAP, and FIC assays.

#### 2.2.1. Free Radical Scavenging Activity with DPPH Assay

The free radical scavenging activity was determined by the 1,1-diphenyl-2-picryl-hydrazyl (DPPH) assay as described by Molyneux et al. [63], with some modifications. To this end, 50 μL of a hydroalcoholic fraction of PLSO (from 0.016 mg/mL to 2 mg/mL) was mixed with 950 μL of a methanolic DPPH solution (10^−4^ M). After incubation for 30 min in the dark, the absorbance was measured at 517 nm. The antioxidant activity related to the DPPH radical scavenging effect was expressed as a percent of inhibition (PI) using the following equation:

PI = [(A0 − A1)/A0] × 100

where A0 is the absorbance of the DPPH solution and A1 is the absorbance of the DPPH solution after the addition of the sample. The antioxidant activity was expressed as IC50. A low IC50 value corresponds to a high antiradical activity. Ascorbic acid was used as the positive control and all tests were carried in triplicate.

#### 2.2.2. Ferric Reducing Antioxidant Power (FRAP) Assay

The ferric reducing antioxidant power (FRAP) was determined according to the method of Bassene et al. [64]. Briefly, 400 μL of a hydroalcoholic fraction of PLSO (from 0.016 mg/mL to 2 mg/mL) was mixed with 1 mL of phosphate buffer (0.2 M; pH 6.6) and 1 mL of 1% potassium hexacyanoferrate (K_3_Fe (CN)_6_). After incubation in a water bath at 50 °C for 30 min in the dark, 1 mL of 10% trichloroacetic acid was added, and the mixture was then centrifuged at 1750× *g* for 10 min. Then, 1 mL of the obtained supernatant was incubated with 200 μL of 0.1% (*w/v*) ferric chloride (FeCl_3_) solution and allowed to stand for 30 min in dark. The absorbance of the reaction mixture was measured spectrophotometrically at 700 nm. Higher value absorbance of the reaction mixture indicated greater reducing power. Ascorbic acid was used as a positive control. The test was carried out in triplicate. The FRAP value of the hydroalcoholic fraction of PLSO was calculated as follows:

FRAP [%] = [(Absorbance_sample_/Absorbance_blank_) × 100/Absorbance_sample_ ]

#### 2.2.3. Ferrous-Ion Chelating (FIC) Assay

The ferrous ion chelating (FIC) activity of PLSO was determined according to the method of Dinis et al. [65], with minor modifications. In total, 100 μL of a hydroalcoholic fraction of PLSO (from 0.016 mg/mL to 2 mg/mL) was mixed with 50 μL of 2 mM FeCl_2_, and 100 μL of 5 mM ferrozine. The mixture was allowed to stand for 10 min at room temperature. The ferrous iron–ferrozine complex formation was then monitored by measuring the absorbance at 562 nm against a blank. Ethylenediaminetetraacetic acid (EDTA) was used as positive control. The assays were performed in triplicate. The percentage of inhibition of ferrozine-Fe ^2+^ complex formation was calculated as below:

FIC (%) = [(Absorbance_negative control_ − Absorbance_sample_) × 100/Absorbance_negative control_]

#### 2.2.4. KRL Test

The overall antioxidant defense potential of the PLSO was measured with the KRL test (Kit Radicaux Libres) [62,66]. This test consists of submitting whole blood to free radical attack to mobilize the radical scavengers present in the blood and to neutralize the oxidation processes [66]. Diluted control blood samples in the presence or absence of PLSO, which was diluted in DMSO, were oxidized by molecular oxygen in an aqueous suspension using a 2.2′–azobis (2-amidinopropane) dihydrochloride (AAPH) solution. Hemolysis was recorded using a 96-well microplate reader (KRL Reader, Kirial International, Lara-Spiral, Couternon, France) by measuring the turbidimetric optical density decay at 620 nm. The antioxidant efficiency of the oil was expressed in Trolox equivalent. The same analysis was conducted with α-tocopherol (Sigma-Aldrich, St-Quentin-Fallavier, France) used as control. The KRL test was realized by the Lara-Spiral laboratory (Couternon, France).

### 2.3. In Vitro Study

#### 2.3.1. Cell Culture and Treatments

Murine C2C12 myoblasts were grown in Dulbecco’s modified Eagle’s medium (DMEM) supplemented with 10% (*v/v*) of heat-inactivated fetal bovine serum (FBS) and 1% (*v/v*) of penicillin (100 U/mL)/streptomycin (100 mg/mL). The cells were maintained in a humidified atmosphere (5% CO_2_, 95% air) at 37 °C. For subcultures, cells were trypsinized (0.05% trypsin − 0.02% EDTA solution) and passed twice a week. 7β-OHC was either from Sigma-Aldrich or provided by Mohammad Samadi (University of Lorraine, Metz, France); the purity was higher than 98%. The stock solutions of 7β-OHC was prepared at 800 µg/mL (2 mM), as previously described by Ragot et al. [67], and stored in the dark at 4 °C. A stock solution of PLSO was prepared at 80 mg/mL in dimethyl sulfoxide (DMSO; Sigma-Aldrich) and stored in the dark at 4 °C. An α-tocopherol (the major component of vitamin E; Sigma-Aldrich) solution was prepared to 80 mM in absolute ethanol, as previously described [60], and stored in the dark at 4 °C. For the different experiments, C2C12 myoblasts were used at 80% confluency; they were seeded either into Petri dishes of 10 cm in diameter (1.2 × 10^6^ cells per Petri dish), in six-well plates (2 × 10^5^ cells per well), or in 96-well plates (10 × 10^4^ cells per well). After 12 h, the growth medium was removed and the C2C12 cells were incubated with 7β-OHC (20 µg/mL/50 µM) for 24 h with or without PLSO (100 µg/mL), or α-tocopherol (400 µM) (used as a positive control for cytoprotection) [28]. PLSO and α-tocopherol were introduced in the culture medium 2 h before 7β-OHC. The choice of the concentration of 7β-OHC (20 µg/mL/50 µM) and PLSO (100 µg/mL) was based on the dose-effect of 7β-OHC (5–80 µg/mL/12.5–200 µM) and PLSO (5–3200 µg/mL), which was realized with an MTT assay.

#### 2.3.2. Evaluation of Cell Morphology by Phase-Contrast Microscopy

After 24 h of treatment with or without 7β-OHC (20 µg/mL/50 µM) in the presence or absence of PLSO (100 µg/mL), or of α-tocopherol (400 µM), the cell morphology and cell density of C2C12 myoblasts cells were observed and photographed using a phase-contrast microscope (Axiovert 40 CFL, Zeiss, Jena, Germany) equipped with a digital camera (Axiocam lCm1, Zeiss).

#### 2.3.3. Evaluation of Cell Viability with the MTT Assay

Cell viability was measured using an MTT (3-(4, 5-dimethylthiazol-2-yl)-2, 5-diphenyltetrazolium bromide) assay. MTT salt is reduced to formazan in metabolically active cells by the mitochondrial enzyme succinate dehydrogenase [35]. C2C12 cells were seeded into 96-well flat-bottom culture plates. After 24 h of treatment as described above, an MTT solution (0.05 mg/mL, dissolved in culture medium) was added to each well and incubated for 3 h at 37 °C. The medium was removed and 100 µL of dimethyl sulfoxide (DMSO) was added to dissolve the formed formazan crystals. The percentage of viable cells was calculated based on a reduction of the MTT dye into formazan crystals at 570 nm using a microplate reader (Tecan Sunrise, Tecan, Lyon, France).

#### 2.3.4. Measurement of Cell Viability with the Fluorescein Diacetate Assay

The fluorescein diacetate (FDA) assay evaluates the ability of living cells to transform the FDA to fluorescein after cleavage by plasma membrane esterases [68]. After 24 h of treatment with or without 7β-OHC (50 µM) in the presence or absence of PLSO (100 µg/mL), α-tocopherol (400 µM), or MitoQ (1 µM) [69,70], C2C12 cells were incubated for 5 min at 37 °C with 50 µM FDA (Sigma-Aldrich) and then lysed with 10 mM of a Tris-HCl solution containing 1% sodium dodecyl sulfate (SDS, Sigma-Aldrich). The fluorescence intensity of the fluorescein (λ_Ex max_ = 485 nm, λ_Em max_ = 528 nm) was measured with a Tecan fluorescence microplate reader (Tecan Infinite M200 Pro, Lyon, France) in order to quantify the living cells. The results were expressed as the % of control: (Fluorescence (assay)/Fluorescence (control)) × 100.

#### 2.3.5. Measurement of Plasma Membrane Permeability with Propidium Iodide

Propidium iodide (PI) was used to evaluate the plasma membrane permeability and cell death. This dye penetrates cells with damaged plasma membranes considered as dead cells [71]. After 24 h of treatment with or without 7β-OHC (50 µM) in the presence or absence of PLSO (100 µg/mL) or α-tocopherol (400 µM), C2C12 cells (adherent and non-adherent cells) were stained with a PI solution (1 µg/mL of PBS) for 5 min at 37 °C, and then immediately analyzed on a BD Accuri™ C6 flow cytometer (BD Biosciences, San Jose, CA, USA). The red fluorescence was selected on a 630 nm band-pass filter and 10,000 cells were acquired for each sample. Data analyses were performed using FlowJo software (Carrboro, NC, USA).

#### 2.3.6. Measurement of Oxidative Stress

##### Evaluation of Reactive Oxygen Species Production with Dihydroethidium

Dihydroethidium (DHE) was used to detect ROS, mainly superoxide anion (O_2_^●−^) production. DHE, a dye that can freely diffuse across cell membranes, is rapidly oxidized under the action of ROS to fluorescent ethidium. This latter exhibits an orange/red fluorescence (λ_Ex max_ = 488 nm; λ_Em max_ = 575 nm) [72]. After 24 h of treatment, C2C12 cells (adherent and non-adherent cells) were stained with a 2 µM DHE solution for 15 min at 37 °C and then analyzed on a BD Accuri™ C6 flow cytometer (BD Biosciences). The fluorescent signals of the DHE-stained cells were collected through a 580 nm band-pass filter and 10,000 cells were acquired for each sample. Data analyses were performed using FlowJo software.

##### Quantification of Antioxidant Enzymes Activities: Glutathione Peroxidase (GPx) and Superoxide Dismutase (SOD)

Glutathione peroxidase (GPx) activity was evaluated according to the method described by Flohe and Günzler [73]. After 24 h of treatment, C2C12 cells were trypsinized, lysed by sonication, and centrifuged at 20,000× *g* (30 min; 4 °C). The supernatant was incubated for 5 min at 25 °C with 0.1 mM of reduced glutathione (GSH) and phosphate buffer saline (50 mM, pH 7.8). The reaction was initiated by the addition of H_2_O_2_ and stopped by cell incubation with trichloroacetic acid, for 30 min on ice. After centrifugation at 1000× *g* for 10 min, the supernatant was transferred into a new tube and 0.32 M of Na_2_HPO_4_·12H_2_O and 1 mM of DTNB were added to the supernatant and the color developed was measured at 412 nm. GPx activity was expressed as the percentage of control cells.

Superoxide dismutase (SOD) activity was measured following the method of Beauchamp and Fridovich [74]. Cell lysates were incubated in the presence of 50 mM phosphate buffer, 0.1 mM EDTA, 13 mM L-methionine, 2 µM riboflavin, and 75 mM nitro bleu tetrazolium (NBT). The mixture was exposed to white light for 20 min. The developed blue color is proportional to SOD activity and was measured at 560 nm. Units of SOD activity are expressed as the amount of enzyme required to inhibit by 50% the reduction in NBT. SOD activity was expressed as a percentage of the controls. Antioxidant enzyme activity was expressed relative to the protein content, determined with a Bradford assay.

##### Measurement of Lipid Peroxidation Products: Malondialdehyde (MDA) and Conjugated Dienes (CDs)

Oxidation of polyunsaturated fatty acid was estimated by the measurement of the final lipid peroxidation products, such as malondialdehyde (MDA) and conjugated dienes (CDs).

Measurement of MDA level: The MDA level was measured using the method described by Yoshioka et al. [75]. Briefly, C2C12 cell lysates were mixed with 1.5 mL of a reactive mixture containing 20% trichloroacetic acid and 0.67% thiobarbituric acid. The samples were incubated for 30 min in a water bath at a temperature of 95 °C. After cooling, 4 mL of n-butanol was added, and the mixture was centrifuged (1600× *g* for 10 min) to remove undissolved materials. Then, the absorbance was measured at 532 nm. The concentration of MDA was expressed as nmol/mg of protein.

Measurement of CDs level: The CDs level was quantified as previously described by Esterbauer et al. [76]. Lipids were extracted from C2C12 cell lysates using a chloroform and methanol mixture (2:1; *v/v*). After vigorous agitation for 2 min, the material was subjected to centrifugation (1200× *g*; 3 min), and the lower layer was aspirated, transferred into a new test tube, and evaporated under a nitrogen atmosphere. The residue was reconstituted with 1 mL of hexane and measured spectrophotometrically at 243  nm. The results were expressed as nmoles hydroperoxide/mg of protein.

Measurement of Protein Oxidation Products: Carbonylated Proteins (CPs)

Carbonylated proteins (CPs) concentration was measured as described by Oliver et al. [77]. This assay is based on the reaction between 2,4-dinitrophenylhydrazine (DNPH) and CPs to form protein hydrazones. Briefly, C2C12 cell lysates were incubated with DNPH (10 mM in 2.5 N HCl) in the dark for 1 h at room temperature. Then, 20% trichloroacetic acid was added for a 10 min incubation time on ice and the tubes were centrifuged at 1600× *g* for 5 min. The protein pellets were washed with 10% trichloroacetic acid and ethanol-ethyl acetate (1:1; *v/v*) mixture to remove free DNPH. The final pellet was dissolved in a 6 M guanidine hydrochloride solution, and the absorbance was read at 370 nm. The concentration of CPs was expressed in nmol/mg of protein.

#### 2.3.7. Evaluation of Mitochondrial Function

##### Measurement of Transmembrane Mitochondrial Potential with DiOC_6_(3)

The variation in the mitochondrial transmembrane potential (ΔΨm) was detected using 3,3′-dihexyloxacarbocyanine iodide (DiOC_6_(3)) [67]. After 24 h of treatment, adherent and non-adherent C2C12 cells were pooled, stained with a solution of DiOC_6_(3) (Invitrogen/Thermo Fisher Scientific, Montigny le Bretonneux, France) at 40 nM (15 min; 37 °C), and then analyzed on a BD Accuri™ C6 flow cytometer (BD Biosciences). The loss of ΔΨm is indicated by a decrease in the green fluorescence intensity collected through a 520 ± 10 nm band-pass filter. For each sample, 10,000 cells were acquired, and data analyses were performed with FlowJo software.

##### Measurement of ATP Levels

The adenosine triphosphate (ATP) assay was performed using the ATP Bioluminescence Assay Kit CLS II (ref # 11699709001, Roche, Meylan, France), according to the manufacturer’s procedure. At the end of the treatments, cells were collected by trypsinization, adherent and non-adherent cells were mixed, and the ATP level was determined after cell lysis. To this end, 100 µL of cell lysis reagent was added on the cell pellets. After 10 min of incubation at RT, a centrifugation was realized at 1000× *g* for 5 min. Then, 50 µL of luciferase was added to 50 µL of each cell lysate to measure the bioluminescence of the samples using a microplate reader (Tecan Infinite M200 Pro). A standard calibration curve was prepared from an ATP stock solution (10.5 mg/mL) using lyophilized ATP provided by the kit to determine the cellular ATP concentration.

##### Measurement of Mitochondrial Reactive Oxygen Species with MitoSOX-Red

Mitochondrial ROS production, including superoxide anion (O_2_^●−^), was measured by flow cytometry after staining with MitoSOX-Red (Thermo Fisher Scientific, Asheville, NC, USA). Once in the mitochondria, this probe is oxidized and exhibits an orange/red fluorescence (λ_Ex max_ = 510 nm; λ_Em max_ = 580 nm) [78]. After 24 h of treatment, adherent and non-adherent C2C12 cells were polled and stained with a 5 mM MitoSOX-Red solution for 15 min at 37 °C and then analyzed on a BD Accuri™ C6 flow cytometer. The fluorescent signals were collected through a 580 ± 20 nm band pass filter. For each sample, 10,000 cells were acquired, and data analyses were performed using FlowJo software.

#### 2.3.8. Determination of the Peroxisomal Status

##### Evaluation of the Level and Topography of Abcd3 Peroxisomal Transporter by Structured Illumination Microscopy (Apotome)

ATP binding cassette subfamily D member (Abcd3) peroxisomal transporter was detected by indirect immunofluorescence [79,80]. Cells were cultured on glass slides in six-well plates. At the end of the treatment, adherent and non-adherent C2C12 cells were collected, fixed with 2% (*w/v*) paraformaldehyde for 15 min at RT, and then rinsed twice with PBS. Cells were permeabilized for 30 min at RT with a PFS buffer (PBS/0.05% saponin/10% FCS). After washing in PBS, cells were incubated (1 h, RT) with an appropriate rabbit polyclonal antibody raised against Abcd3 (# 11523651, Pierce/Thermo Fisher Scientific, Asheville, NC, USA) diluted (1/500) in PFS buffer. Cells were washed and incubated in the dark (30 min, RT) with a goat anti-rabbit 488-Alexa antibody (Santa-Cruz Biotechnology, Santa Cruz, CA, USA) diluted at 1/500 in PFS buffer. After washing in PBS, cells were stained with Hoechst 33342 (1 µg/mL) and then mounted in Dako fluorescent mounting medium (Dako, Copenhagen, Denmark). The slides were stored in the dark at 4 °C and examined with structured illumination microscopy (Apotome). The images were realized with ZEN imaging software (Zeiss).

##### Flow Cytometric Quantification of Abcd3 Peroxisomal Transporter

For flow cytometric analyses, adherent and non-adherent C2C12 cells were collected, fixed with 2% (*w*/*v*) paraformaldehyde diluted in PBS for 15 min at RT and then rinsed twice with PBS. Cells were permeabilized for 30 min at RT with PFS buffer. After washing in PBS, cells were incubated (1 h, RT) with an appropriate rabbit polyclonal antibody raised against Abcd3 (# 11523651, Pierce/Thermo Fisher Scientific) diluted (1/500) in PFS buffer. Cells were washed and incubated in the dark (30 min, RT) with a goat anti-rabbit 488-Alexa antibody (Santa-Cruz Biotechnology, Santa Cruz, CA, USA) diluted at 1/500 in a PFS buffer. After washing in PBS, cells were resuspended in PBS and immediately analyzed on a BD Accuri™ C6 flow cytometer (BD Biosciences). The green fluorescence of 488-Alexa was collected with a 520 ± 20 nm band-pass filter. For each sample, 10,000 cells were acquired, and data analyses were performed using FlowJo software.

##### Gas Chromatography—Mass Spectrometry Analysis of Fatty Acids

Fatty acids, including very-long-chain fatty acids (VLCFA; C ≥ 22) [81], were analyzed using gas chromatography coupled to mass spectrometry (GC-MS), as previously described by Blondelle et al. [82]. Total cellular lipids were extracted, according to the method of Folch et al. [83]. Fatty acids were quantitated by calculating the relative response ratios to their closest internal standard. Calibration curves were obtained with fatty acid authentic standards processed as cell pellets.

##### Transmission Electron Microscopy Analysis

At the ultrastructural level, transmission electron microscopy (TEM) is the most powerful tools to observe morphological changes caused by various physical or chemical agents [84]. TEM was used to visualize mitochondrial and peroxisomal changes [80] in C2C12 cells cultured for 24 h in the presence or absence of 7β-OHC (50 µM) without or with PLSO (100 µg/mL) or α-tocopherol (400 µM) [85]. The samples were fixed for 1 h at 4 °C in 2.5% (*w/v*) glutaraldehyde diluted in a cacodylate buffer (0.1 M, pH 7.4), washed twice in cacodylate buffer, incubated in the dark for 1 h at 21 °C in Tris–HCl (0.05 M, pH 9.0) containing diaminobenzidine (DAB: 2.5 mg/mL) and H_2_O_2_ (10 µL/mL of a 3% solution); washed in cacodylate buffer (0.1 M, pH 7.4) for 5 min at 21 °C; post-fixed in 1% (*w/v*) osmium tetroxide diluted in cacodylate sodium (0.1 M, pH 7.4) for 1 h at 21 °C in the dark; and rinsed in cacodylate buffer (0.1 M, pH 7.4). The preparations were dehydrated in graded ethanol solutions and then embedded in Epon. Ultrathin sections were cut with an ultramicrotome, contrasted with uranyl acetate and lead citrate, and examined using an HT7800 electron microscope (Hitachi, Tokyo, Japan) operating at 100 kV and equipped with two advanced microscopy technique (AMT) cameras (Woburn, MA, USA).

##### Analysis of Abcd3 Peroxisomal Transporter mRNA by Real-Time Quantitative Polymerase Chain Reaction

Total mRNA from C2C12 cells were extracted and purified using the RNeasy Mini Kit (Qiagen, Courtaboeuf, France). Total mRNA concentration was measured with TrayCell (Hellma, Paris, France) and the purity of the nucleic acids was controlled by the ratio of absorbance at 260 nm and 280 nm (ratios between 1.8 and 2.2 were considered satisfactory). One microgram of total mRNA from each sample was converted into single-stranded cDNA using the iScript cDNA Synthesis kit (BioRad, Marne la Coquette, France) according to the following procedure: 5 min at 25 °C, 20 min at 46 °C, and 5 min at 95 °C. cDNA was then amplified using the Takyon TM Rox SYBR Master Mix dTTP Blue (Eurogentec, Liège, Belgium) and 300 nM of forward and reverse mouse Abcd3 primer. The forward and reverse Abcd3 primer sequences were the following: Forward: 5′-ctgggcgtgaaatgactagattg-3′; Reverse 5′-cttctcctgttgtgacaccattg-3′.

Thermal cycling conditions were as follows: activation of DNA polymerase (95 °C, 10 min), followed by 40 cycles of amplification at 95 °C for 15 s, 60 °C for 30 s, and 72 °C for 30 s, followed by a melting curve analysis to control for the absence of non-specific products. Gene expression was quantified using cycle to threshold (Ct) values and normalized by the 36B4 reference gene (Forward: 5′-gcgacctggaagtccaacta-3′; Reverse: 5′-atctgcttggagcccacat-3′). Abcd3 level was determined as fold induction of the control.

### 2.4. Gas Chromatography—Mass Spectrometry Analysis of Cholesterol and Oxysterols Oxidized at C7 (7-Ketocholesterol, 7β-Hydroxycholesterol) in the Plasma of Sarcopenic Patients

Cholesterol and oxysterols levels (7KC, 7β-OHC) were determined by GC-MS on plasma samples from Tunisian subjects. All subjects gave their written consent before being enrolled in this preliminary study. In total, 45 adults of 65 years and older (23 men, 22 women) were recruited over a period of 1 month from January to February 2019. All participants were recruited from a nursing home (Sousse, Tunisia). The Timed Up and Go (TUG) test was used to classify patients as sarcopenic (22 subjects; age = 80 ± 4.16; female/male = 15/7) and non-sarcopenic (23 subjects; age = 70.84 ± 4.38; female/male = 7/16). Blood samples were collected in EDTA tubes after overnight fasting. The blood samples were centrifuged at 800× *g* (10 min; 4 °C), and the plasma was divided into several aliquots that were immediately frozen at −80 °C and stored for one year until GC-MS analysis. Oxysterols were quantified as follows: in a glass tube, 300 μL of plasma was suspended in absolute ethanol containing BHT (50 μg/mL). 7KC (d7) and 7β-OHC (d7) (Avanti Polar Lipids, 700 Industrial Park Drive Alabaster, AL, USA) were used as internal standards. Samples were subjected to alkaline hydrolysis with 10 M KOH (1 h; 37 °C). The reaction mixture was washed with water in order to adjust to pH 7 and sterols were extracted with hexane. After solvent evaporation, 100 μL of a mixture of pyridine/hexamethyldisilazane (HMDS)/trimethylchlorosilane (TMCS) (3:2:1; *v/v/v*) (Acros Organics, Fisher Scientific, Asheville, NC, USA) was added, and samples were incubated at 60 °C for 30 min to form trimethylsilyl ethers. After evaporation, the residue was dissolved in 100 μL hexane for GC-MS analysis. GC-MS was performed using an Agilent Technology 6890 GC equipped with an HP7683 injector and a 5973-mass selective detector (Agilent Technologies, Santa Clara, CA, USA). Chromatography was performed using an HP-5MS-fused silica capillary column (length: 25 m; i.d.: 0.25 mm; film thickness: 0.25 μm; Agilent Technologies, Santa Clara, CA, USA). GC-MS conditions were as follows: carrier gas, helium at a flow rate of 1.1 mL/min; injector temperature, 250 °C; oven temperature180 °C, which increased at 10 °C/min to 260 °C, then at 1 °C/min to 280 °C and held for 5 min. The mass spectrometer was operated in the electron impact mode with electron energy of 70 eV. The ion source temperature and the quadrupole temperature were 230 °C and 150 °C, respectively. The ions used for analysis were 7β-OHC 456 *m*/*z*, 7β-OHC (d7) 463 *m*/*z*, and 7KC 472 *m*/*z*, 7KC (d7) 479 *m*/*z*. Calibration curves were obtained using authentic standards extracted with the method used for the samples.

### 2.5. Statistical Analysis

The experimental results were statistically analyzed with GraphPad Prism 8.0 software (GraphPad Software, San Diego, CA, USA). In vitro data were expressed as the mean ± standard deviation (SD) and compared with an ANOVA test followed by a Tukey’s test, which allows multiple comparisons and permits to assess any interaction. Clinical data were compared with a Student’s *t*-test. A *p*-value less than 0.05 was considered statistically significant. The heatmap representation was realized with GraphPad Prism 8.0 software.

## 3. Results

### 3.1. Biochemical Composition of Pistacia lentiscus *L*. Seed Oil

The profiles of polyphenols, flavonoids, and carotenoids contents in *Pistacia lentiscus* L. seed oil (PLSO) were measured using colorimetric methods and the results are shown in Table 1. The amounts of total phenols, flavonoids, and carotenoids in PLSO are 28.50 ± 0.77 gallic acid equivalents (mg GAE/g of extract), 51.36 ± 2.30 catechin equivalent (mg CE/g of extract), and 2083.59 ± 55.00 (mg/kg of extract), respectively.

In PLSO, the polyphenols identified and characterized by HPLC coupled with UV analysis were protocatechuic acid, which is a dihydroxybenzoic acid and a type of phenolic acid, and coumarin, which belongs to a polyphenol subclass (hydroxycoumarins) (Table 2).

The fatty acid profile of PLSO, expressed as percentage of total fatty acids, was determined using GC and the results are presented in Table 3. The main fatty acids detected in PLSO were oleic acid (49.77 ± 0.12%), palmitic acid (27.20 ± 0.22%), and linoleic acid (17.19 ± 0.10%), followed by palmitoleic acid (2.25 ± 0.01%), vaccenic acid (1.51 ± 0.03%), and stearic acid (1.26 ± 0.01%). Likewise, PLSO also contains α-linolenic acid, gadoleic acid, and arachidic acid but in much smaller quantities (0.12–0.39%). Minor monounsaturated fatty acids, such as myristic acid, margaric acid, lignoceric acid, and behenic acid, were also detected, but in trace amounts (≤0.05%).

The sterol composition of PLSO was determined using GC and spectral analysis and the results are shown in Table 4. The most abundant detected phytosterols in PLSO were β-sitosterol (67.25 ± 3.24 mg/100 g oil), α-epoxysitostanol (33.36 ± 1.65 mg/100 g oil), and 24-methylene cycloartenol (16.10 ± 2.72 mg/100 g oil) followed by cycloartenol (9.35 ± 1.49 mg/100 g oil) and campestanol (4.48 ± 0.85 mg/100 g oil). All other phytosterols were present in small amounts.

The α-tocopherol content of PLSO is given in Table 5. The results show that α-tocopherol represented 68.1 ± 3.41 mg/kg of oil.

### 3.2. Evaluation of the Antioxidant Properties of Pistacia lentiscus *L*. Seed Oil

The antioxidant activities of PLSO were measured with different assays: DPPH, FRAP, FIC, and KRL. The results are shown in Table 6. PLSO exhibits free radical scavenging activity, as shown by the IC50 value (5.01 ± 0.095 mg/mL). The half-maximal inhibitory concentration (IC50) (volume of oil required to lower the initial DPPH concentration by 50%) was determined from the dose–response curve. This activity was less than those of ascorbic acid (AA) used as the standard.

The reducing power of PLSO measured by FRAP assay was investigated along with AA used as the standard reference. The IC50 value was 1.15 ± 0.23 mg/mL (Table 6). In addition, an iron-chelating activity evaluated with the FIC assay was observed in PLSO with an IC50 of 5.61 ± 0.14 mg/mL (Table 6).

The antioxidant properties of α-tocopherol, PLSO, and Argan Oil Roasted Agadir (AO) were evaluated with the KRL test. For the PLSO and AO, the antioxidant activities were expressed in Trolox equivalent (mole Trolox/mL of oil). For the KRL test, we used α-tocopherol as the positive control and AO stored for 5 years at 4 °C in the dark as the negative control (the corresponding AO freshly prepared has been previously characterized and described and was strongly antioxidant [86]). PLSO showed a higher KRL antioxidant status than the 5 years stored AO. KRL values were 4440.00 ± 493.60 and 360.8 ± 153.6 (Trolox equivalent) in PLSO and AO, respectively (Table 6), illustrating that after 5 years of storage, AO shows a notable decrease in antioxidant activity.

### 3.3. Evaluation of 7-Ketocholesterol and 7β-Hydroxycholesterol Plasma Levels in Sarcopenic and Non-Sarcopenic Subjects

As several studies support that 7KC and 7β-OHC, mainly resulting from cholesterol auto-oxidation, are involved in the development of major age-related diseases [31,87], these oxysterols as well as cholesterol were measured by GC-MS in the plasma of non-sarcopenic and sarcopenic subjects. In sarcopenic patients, the 7β-OHC level was significantly higher than in non-sarcopenic subjects whereas no significant difference in 7KC and cholesterol level was observed (Supplementary Figure S5). This plasma increase in 7β-OHC could favor the accumulation of this oxysterol in skeletal muscle. Indeed, in male Wistar rats in response to chronic alcohol feeding, there were significant increases in the soleus (type I fiber, glycolytic, aerobic activity) of 7α-OHC, 7β-OHC, and 7KC, whereas in the plantaris (type II fiber, anaerobic activity) only 7β-OHC was increased [88]. Furthermore, in zebra fish, 25-hydroxycholesterol alters muscle morphology and reduces mobility; a similar effect can be envisaged with 7β-OHC [89]. Based on this previous works, and knowing that lipotoxicity (defined as an abnormal accumulation of lipids in tissues such as skeletal muscles) leads to metabolic and functional dysfunctions [90], it is important to clarify whether 7β-OHC can have cytotoxic effects on skeletal muscle cells, and if so, to find treatments to counteract this toxicity. To evaluate this hypothesis, the C2C12 murine myoblast model cultured in the presence of 7β-OHC in the presence or absence of PLSO was used.

### 3.4. Evaluation of the Effects of Pistacia lentiscus *L*. Seed Oil on 7β-Hydroxycholesterol-Induced Morphological Changes and Cell Death

At first, initial experiments were performed on C2C12 cells to evaluate whether PLSO and 7β-OHC alone induce cell death on murine C2C12 myoblast cells. To this end, C2C12 were incubated with various concentrations of PLSO (5 to 3200 µg/mL) for 24 h and cell viability was determined using an MTT assay. As shown in Supplementary Figure S6A, no cytotoxic effects of PLSO (5–800 µg/mL) were observed compared to untreated cells; however, in the presence of PLSO used at 1600 μg/mL and 3200 μg/mL, the percentage of viable cells decreased from 50 to 70%, respectively.

To define whether 7β-OHC was able to influence C2C12cell viability, C2C12 cells were exposed to various concentrations of 7β-OHC (12.5 to 200 µM) for 24 h. In the presence of 7β-OHC (50 µM), cell viability significantly decreased to 48.4% compared to untreated cells (Supplementary Figure S6B). Based on these results, PLSO (100 µg/mL) and 7β-OHC (50 µM) were selected to perform further experiments.

Thus, C2C12 cells were incubated for 24 h with or without 7β-OHC (50 µM) in the presence or absence of PLSO (100 µg/mL) or α-tocopherol (400 µM) used as positive control for cytoprotection. PLSO and α-tocopherol were added 2 h prior 7β-OHC. Based on the observations performed by phase-contrast microscopy, morphological changes in C2C12 cells were observed under treatment with 7β-OHC. In 7β-OHC-treated C2C12 cells, compared to untreated cells, an increased number of round cells floating in the culture medium were observed, reflecting a loss of cell adhesion and an induction of cell death; a reduced number of adherent cells was also observed This effect was remarkably corrected when the cells were simultaneously incubated with PLSO (100 µg/mL) or α-tocopherol (400 µM), indicating that PLSO and α-tocopherol provided protection against 7β-OHC-induced loss of cell adhesion and cell death (Supplementary Figure S7A). By phase-contrast microscopy, no effects on cell adhesion and cell growth of the different vehicles used were observed (Supplementary Figure S7B).

**Figure 1:**
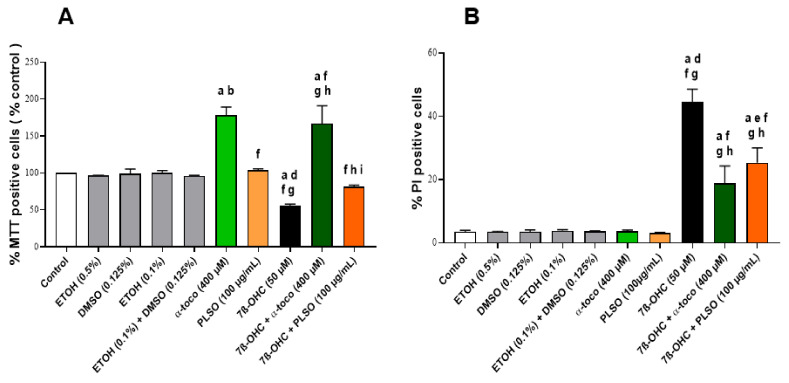
**Effect of *Pistacia lentiscus* L. seed oil** (**PLSO**) **and 7β-hydroxycholesterolin C2C12 cell viability.** C2C12 cells were incubated for 24 h with or without 7β-OHC (50 µM) in the presence or absence of PLSO (100 µg/mL) or α-tocopherol (400 µM). The protective effect of PLSO and α-tocopherol against 7β-OHC-induced cell death was evaluated with the MTT assay (**A**) and by flow cytometry after staining with propidium iodide (PI) (**B**). Data are the mean ± SD of two independent experiments performed in triplicate. A multiple comparative analysis between the groups, taking into account the interactions, was carried out using an ANOVA test followed by a Tukey’s test. A *p*-value less than 0.05 was considered statistically significant. The statistically significant differences between the groups, which are indicated by different letters, take into account the vehicle used. a: comparison versus control; b: comparison versus ETOH (0.5%); c: comparison versus DMSO (0.125%); d: comparison versus ETOH (0.1%); e: comparison versus (ETOH (0.1%) + DMSO (0.125%)); f: comparison versus α-toco (400 µM); g: comparison versus PLSO (100 µg/mL); h: comparison versus 7β-OHC (50 µM); i: comparison versus 7β-OHC (50 µM) + α-toco (400 µM). No significant differences were observed between the untreated (control) and vehicle-treated cells: EtOH (0.5%), DMSO (0.125%), EtOH (0.1%), and EtOH (0.1%) +DMSO (0.125%).

The cytoprotective effect of PLSO (100 µg/mL) was confirmed with the MTT assay. As shown in Figure 1A, the percentage of MTT-positive cells, reflecting metabolically active cells, was significantly decreased in the 7β-OHC (50 µM)-treated cells compared to the control. Noteworthy, when PLSO (100 µg/mL) was associated with 7β-OHC (50 µM), the percentage of MTT-positive cells was significantly increased: this demonstrates that PLSO attenuates 7β-OHC-induced cell death. Similar results were obtained with α-tocopherol.

To further investigate the effect of PLSO on plasma membrane permeability and/or cell death, staining with propidium iodide (PI) was used. As illustrated in Figure 1B, the percentage of PI-positive cells was significantly increased after exposure to 7β-OHC (50 µM), indicating that this oxysterol caused altered plasma membrane and/or cell death in C2C12 cells. In the presence of PLSO (100 µg/mL) or α-tocopherol (400 µM) associated with 7β-OHC (50 µM), the percentage of PI-positive cells was significantly decreased compared to 7β-OHC-treated cells. Comparatively to untreated cells, no effect of PLSO (100 µg/mL) or α-tocopherol (400 µM) alone was observed on plasma membrane permeability. Altogether, these data show that PLSO as well as α-tocopherol strongly attenuate 7β-OHC-induced C2C12 cell death.

### 3.5. Evaluation of the Effects of Pistacia lentiscus *L*. Seed Oil on 7β-Hydroxycholesterol-Induced Oxidative Stress

**Figure 2:**
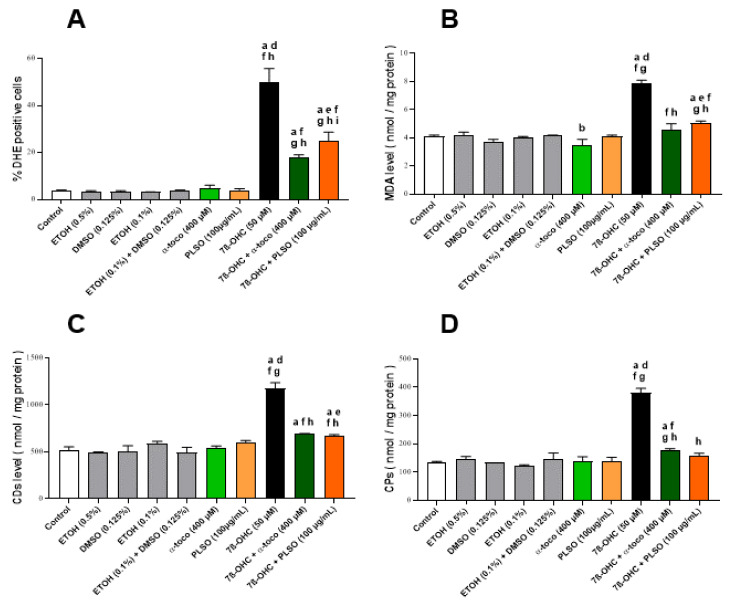
**Effect of Pistacia lentiscus L. seed oil (PLSO) on 7β-hydroxycholesterol-induced overproduction of reactive oxygen species (ROS) and lipid and protein oxidation products in C2C12 cells**. C2C12 cells were incubated for 24 h with or without 7β-OHC (50 µM) in the presence or absence of PLSO (100 µg/mL) or α-tocopherol (400 µM). ROS overproduction was measured by flow cytometry after staining with dihydroethidium (DHE) (**A**). Lipid and protein oxidation products were determined with malondialdehyde (MDA) (**B**), conjugated dienes (CDs) (**C**) and carbonylated proteins (CPs) levels (**D**). Data are presented as the mean ± SD of two independent experiments performed in triplicate. A multiple comparative analysis between the groups, taking into account the interactions, was carried out using an ANOVA test followed by a Tukey’s test. A *p*-value less than 0.05 was considered statistically significant. The statistically significant differences between the groups, which are indicated by different letters, take into account the vehicle used. a: comparison versus control; b: comparison versus ETOH (0.5%); c: comparison versus DMSO (0.125%); d: comparison versus ETOH (0.1%); e: comparison versus (ETOH (0.1%) + DMSO (0.125%)); f: comparison versus α-toco (400 µM); g: comparison versus PLSO (100 µg/mL); h: comparison versus 7β-OHC (50 µM); i: comparison versus 7β-OHC (50 µM) + α-toco (400 µM). No significant differences were observed between the untreated (control) and vehicle-treated cells.

To study the effect of PLSO (100 µg/mL) against 7β-OHC (50 µM)-induced oxidative stress, we measured the production of reactive oxygen species (ROS), lipid peroxidation products (MDA, CDs), carbonylated proteins (CPs), and antioxidant enzyme activities (SOD, GPx) (Figure 2).

ROS overproduction was quantified by flow cytometry with DHE. As shown in Figure 2A, treatment with 7β-OHC induced a significant increase in the percentage of DHE-positive cells compared to the untreated (control) and vehicle-treated (EtOH 0.1%) cells; this increase in ROS production was significantly attenuated when 7β-OHC was associated with PLSO or α-tocopherol.

In addition, the levels of MDA, CDs, and CPs, which are the main products of lipid and protein oxidation, respectively, were significantly higher in 7β-OHC-treated cells compared to untreated (control) or vehicle-treated (EtOH 0.1%) cells; these increases were significantly reduced when the cells were incubated with 7β-OHC in the presence of PLSO or α-tocopherol, comparatively to 7β-OHC (Figure 2B–D).

**Figure 3:**
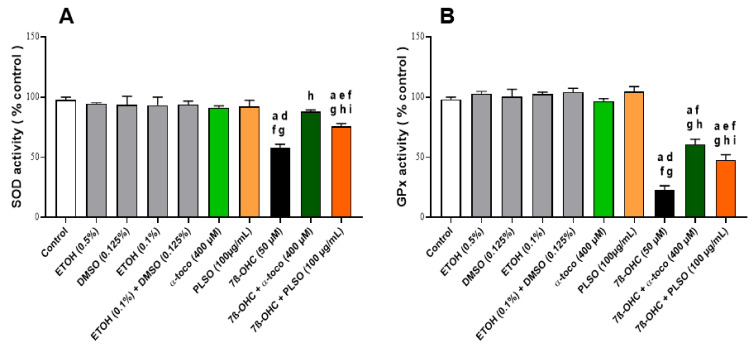
**Effect of Pistacia lentiscus L. seed oil (PLSO) and 7β-hydroxycholesterol on antioxidant enzyme activities (SOD, GPx) in C2C12 cells**. C2C12 cells were incubated for 24 h with or without 7β-OHC (50 µM) in the presence or absence of PLSO (100 µg/mL) or α-tocopherol (400 µM). The measurement of superoxide dismutase (SOD) activity (**A**) and glutathione peroxidase (GPx) activity (**B**) were realized. Data are presented as the mean ± SD of two independent experiments performed in triplicate. A multiple comparative analysis between the groups, taking into account the interactions, was carried out using an ANOVA test followed by a Tukey’s test. A *p*-value less than 0.05 was considered statistically significant. The statistically significant differences between the groups, which are indicated by different letters, take into account the vehicle used. a: comparison versus control; b: comparison versus ETOH (0.5%); c: comparison versus DMSO (0.125%); d: comparison versus ETOH (0.1%); e: comparison versus (ETOH (0.1%) + DMSO (0.125%)); f: comparison versus α-toco (400 µM); g: comparison versus PLSO (100 µg/mL); h: comparison versus 7β-OHC (50 µM); i: comparison versus 7β-OHC (50 µM) + α-toco (400 µM). No significant differences were observed between the untreated (control) and vehicle-treated cells.

In another hand, superoxide dismutase (SOD) and glutathione peroxidase (GPx) activities were significantly decreased in 7β-OHC-treated C2C12 cells when compared with untreated (control) and vehicle-treated (EtOH 0.1%) cells; these decreases were also significantly attenuated when 7β-OHC was associated with PLSO or α–tocopherol (Figure 3).

### 3.6. Evaluation of the Effects of Pistacia lentiscus *L*. Seed Oil on 7β-Hydroxycholesterol-Induced Mitochondrial Damages

**Figure 4:**
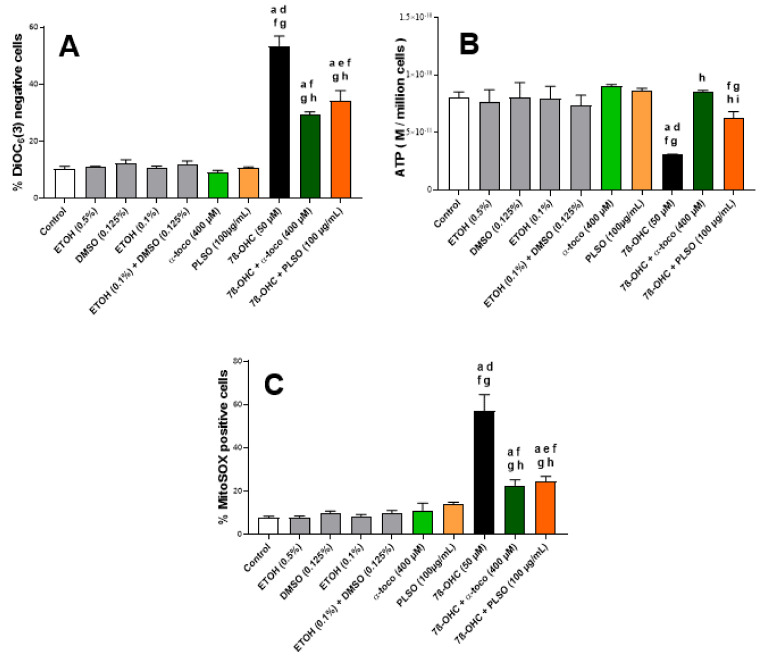
**Effect of *Pistacia lentiscus* L. seed oil (PLSO) on 7β-hydroxycholesterol-induced mitochondrial damage in C2C12 cells.** C2C12 cells were incubated for 24 h with or without 7β-OHC (50 µM) in the presence or absence of PLSO (100 µg/mL) or α-tocopherol (400 µM). Mitochondrial transmembrane potential (∆Ψm) (**A**), mitochondrial ATP production (**B**), and mitochondrial production of superoxide anion(O_2_^●−^) (**C**) were measured. The data are presented as the mean ± SD of two independent experiments performed in triplicate. A multiple comparative analysis between the groups, taking into account the interactions, was carried out using an ANOVA test followed by a Tukey’s test. A *p*-value less than 0.05 was considered statistically significant. The statistically significant differences between the groups, which are indicated by different letters, take into account the vehicle used. a: comparison versus control; b: comparison versus ETOH (0.5%); c: comparison versus DMSO (0.125%); d: comparison versus ETOH (0.1%); e: comparison versus (ETOH (0.1%) + DMSO (0.125%)); f: comparison versus α-toco (400 µM); g: comparison versus PLSO (100 µg/mL); h: comparison versus 7β-OHC (50 µM); i: comparison versus 7β-OHC (50 µM) + α-toco (400 µM). No significant differences were observed between the untreated (control) and vehicle-treated cells.

To evaluate the effect of PLSO (100 µg/mL) against 7β-OHC (50 µM)-induced mitochondrial dysfunction, we measured the mitochondrial transmembrane potential (ΔΨm) after staining with DiOC_6_(3), the overproduction of ROS at the mitochondrial level after staining with MitoSOX-Red, as well as the ATP level by bioluminescence (Figure 4).

Under treatment with 7β-OHC, and comparatively to the untreated (control) and vehicle-treated (EtOH 0.1%) cells, a marked decrease in ∆Ψm, revealed by an increase in the percentage of cells with depolarized mitochondria (DiOC_6_(3)-negative cells), was observed (Figure 4A). In addition, a reduction in ATP production was observed under treatment with 7β-OHC (Figure 4B). With MitoSOX-Red, a marked increase in MitoSOX-Red-positive cells was revealed, confirming a disturbed oxidative phosphorylation and the induction of mitochondrial damage under treatment with 7β-OHC (Figure 4C). Interestingly, in the presence of PLSO or α-tocopherol, the loss of ΔΨm, decrease in ATP, as well as overproduction of mitochondrial ROS was strongly attenuated (Figure 4A–C). In the presence of MitoQ (1 µM), which blocks ROS overproduction at the mitochondrial level, a slight but not significant cytoprotective effect was observed with the FDA test whereas a significant and marked cytoprotection was found with PLSO and α-tocopherol (Supplementary Figure S8), supporting (i) that mitochondrial targeting with an antioxidant is not sufficient to prevent 7β-OHC-induced cell death; and (ii) that PLSO and α-tocopherol, which act at the mitochondrial levels, also have other cellular targets.

### 3.7. Evaluation of the Effects of Pistacia lentiscus *L*. Seed Oil on 7β-Hydroxycholesterol-Induced Peroxisomal Damages

**Figure 5:**
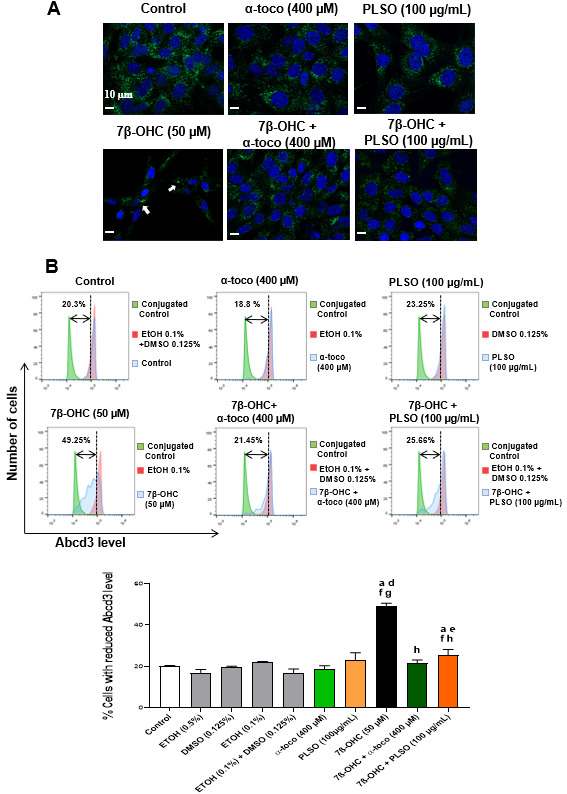
**Effect of Pistacia lentiscus L. seed oil (PLSO) and 7β-hydroxycholesterol on the expression of the major peroxisomal membrane transporter (Abcd3) used to evaluate the peroxisomal topography and mass**. C2C12 cells were incubated for 24 h with or without 7β-OHC (50 µM) in the presence or absence of PLSO (100 µg/mL) or α-tocopherol (400 µM). The protective effect of PLSO and α-tocopherol (400 µM) against 7β-OHC were analyzed by structured illumination microscopy (apotome) (**A**) and flow cytometry (**B**). The white arrows point towards cells with an accumulation of peroxisomes in a particular area of the cytoplasm. The data are presented as the mean ± SD of two independent experiments performed in triplicate. A multiple comparative analysis between the groups, taking into account the interactions, was carried out using an ANOVA test followed by a Tukey’s test. A *p*-value less than 0.05 was considered statistically significant. The statistically significant differences between the groups, which are indicated by different letters, take into account the vehicle used. a: comparison versus control; b: comparison versus ETOH (0.5%); c: comparison versus DMSO (0.125%); d: comparison versus ETOH (0.1%); e: comparison versus (ETOH (0.1%) + DMSO (0.125%)); f: comparison versus α-toco (400 µM); g: comparison versus PLSO (100 µg/mL); h: comparison versus 7β-OHC (50 µM); i: comparison versus 7β-OHC (50 µM) + α-toco (400 µM). In addition, no significant differences were observed between the untreated (control) and vehicle-treated cells.

Abcd3 (ATP binding cassette subfamily D member) is a major component of the peroxisomal membrane and a common constituent of peroxisomes in different tissues [91,92] (Supplementary Figure S9). This peroxisomal transporter is frequently used to evaluate the peroxisomal mass, thus providing information on peroxisome biogenesis [79]. The effect of 7β-OHC (50 µM) with and without PLSO (100 µg/mL) and α-tocopherol (400 µM) was determined on the topography and expression of Abcd3 revealed by indirect immunofluorescence using structured illumination microscopy (Apotome) and flow cytometry (Figure 5).

Using structure illumination microscopy (Figure 5A), a high density of peroxisomes was observed in the cytoplasm of untreated (control) cells, PLSO-, and α-tocopherol-treated cells. An important decrease in peroxisomal density was revealed under treatment with 7β-OHC. In addition, the peroxisomes were homogeneously distributed in the cytoplasm of the control and vehicle-treated cells as well as of PLSO- and α-tocopherol-treated cells, whereas they were preferentially amassed in a particular area of the cytoplasm in 7β-OHC-treated cells. When the cells were cultured in the presence of 7β-OHC associated with PLSO or α-tocopherol, the aspect of the peroxisome in the cytoplasm evocate those of untreated (control) and vehicle-treated cells, although the peroxisomal density remains lower.

Under treatment with 7β-OHC, the decrease in peroxisomal density observed by microscopy suggests a decrease in peroxisomal biogenesis. To confirm this hypothesis, flow cytometric analyses were performed (Figure 5B). The analysis of Abcd3 levels in C2C12 cells did not reveal any difference between untreated (control) and α-tocopherol-treated cells; a slight increase was observed in the presence of PLSO. Under treatment with 7β-OHC, a significant increase in the percentage of cells with a reduced Abcd3 level was observed. Interestingly, this decreased expression of Abcd3 was significantly inhibited when 7β-OHC was combined with PLSO or α-tocopherol.

**Figure 6:**
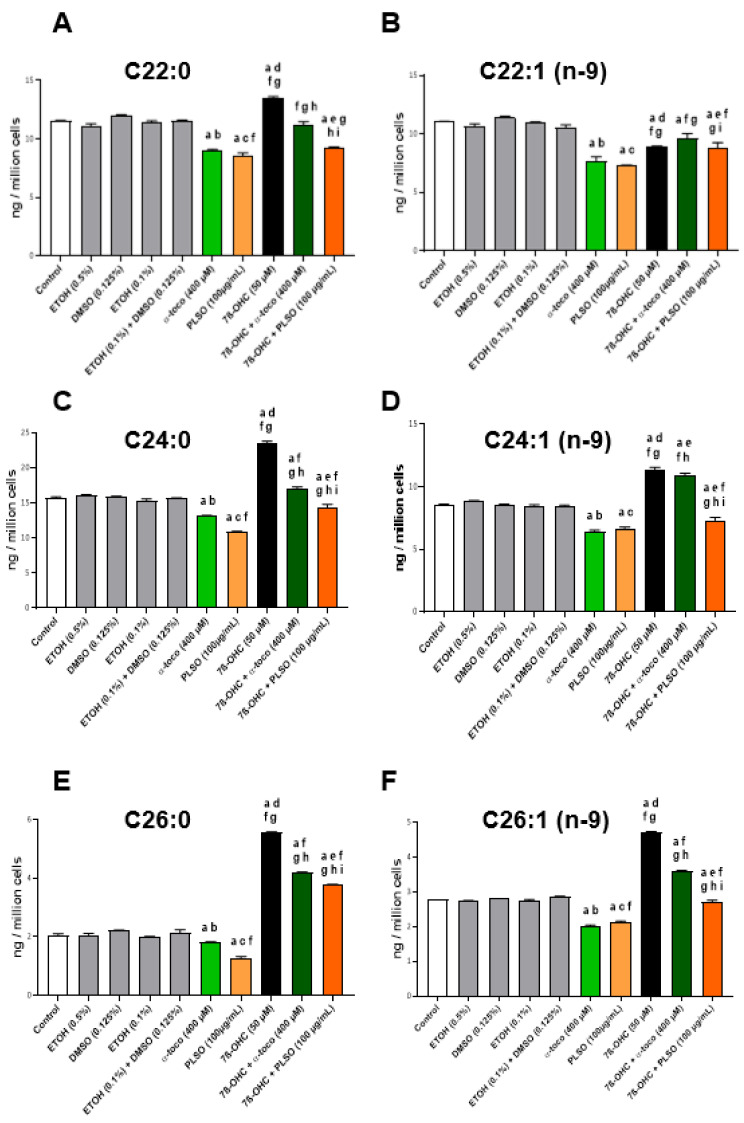
**Effect of 7β-hydroxycholesterol with and without *Pistacia lentiscus* L. seed oil (PLSO) on very-long-chain fatty acid (VLCFA) levels**. C2C12 cells were incubated for 24 h with or without 7β-OHC (50 µM) in the presence or absence of PLSO (100 µg/mL) or α-tocopherol (400 µM). The level of VLCFA (C ≥ 22) was determined by GC-MS: C22:0 (**A**), C22:1 n−9 (**B**), C24:0 (**C**), C24:1 n−9 (**D**), C26:0 (**E**) and C26:1 n−9 (**F**). Data are the mean ± SD of two independent experiments. A multiple comparative analysis between the groups, taking into account the interactions, was carried out using an ANOVA test followed by a Tukey’s test. A p-value less than 0.05 was considered statistically significant. The statistically significant differences between the groups, which are indicated by different letters, take into account the vehicle used. a: comparison versus control; b: comparison versus ETOH (0.5%); c: comparison versus DMSO (0.125%); d: comparison versus ETOH (0.1%); e: comparison versus (ETOH (0.1%) + DMSO (0.125%)); f: comparison versus α-toco (400 µM); g: comparison versus PLSO (100 µg/mL) ; h: comparison versus 7β-OHC (50 µM); i: comparison versus 7β-OHC (50 µM) + α-toco (400 µM). No significant differences were observed between the untreated (control) and vehicle-treated cells.

With regard to peroxisome function, peroxisomal damages (alteration of peroxisomal β-oxidation) (Supplementary Figure S9) can favor the accumulation of very-long-chain fatty acids (VLCFA; C ≥ 22) [81], which can contribute to amplify cell dysfunctions [93]. Therefore, we determined the effect of 7β-OHC (50 µM) associated or not with PLSO (100 µg/mL) or α-tocopherol (400 µM) on VLCFA levels in C2C12 cells. In untreated cells (control) and the vehicle, no significant differences were found; similar levels of VLCFA (C22:0, C24:0, C24:1 n−9, C26:0, and C26:1 n−9) were observed (Figure 6). When C2C12cells were exposed to 7β-OHC, a significant increase in VLCFA was detected, and the latter was significantly reduced when 7β-OHC was associated with PLSO or α-tocopherol (Figure 6).

**Figure 7:**
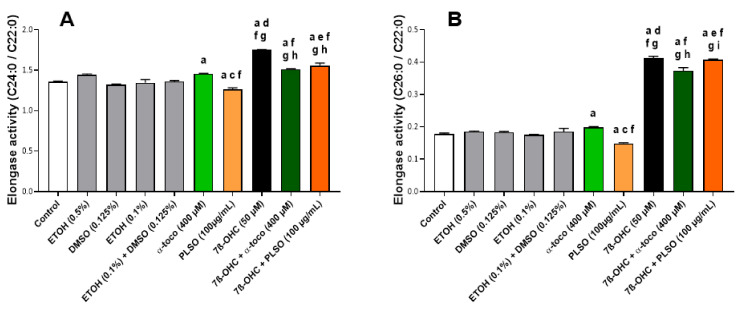
**Effect of *Pistacia lentiscus* L. seed oil** (**PLSO**) **and 7β-hydroxycholesterol on elongase activities.** C2C12 cells were incubated for 24 h with or without 7β-OHC (50 µM) in the presence or absence of PLSO (100 µg/mL) or α-tocopherol (400 µM). The levels of C22:0, C24:0, and C26:0 were determined by GC-MS, and the corresponding elongase activity index, which could correspond to ELOVL1, 3, and 6 activity (ratio (C24:0/C22:0) (**A**) and ratio (C26:0/C22:0) (**B**)) were calculated. Data are the mean ± SD of two independent experiments. A multiple comparative analysis between the groups, taking into account the interactions, was carried out using an ANOVA test followed by a Tukey’s test. A *p*-value less than 0.05 was considered statistically significant. The statistically significant differences between the groups, which are indicated by different letters, take into account the vehicle used. a: comparison versus control; b: comparison versus ETOH (0.5%); c: comparison versus DMSO (0.125%); d: comparison versus ETOH (0.1%); e: comparison versus (ETOH (0.1%) + DMSO (0.125%)); f: comparison versus α-toco (400 µM); g: comparison versus PLSO (100 µg/mL); h: comparison versus 7β-OHC (50 µM); i: comparison versus 7β-OHC (50 µM) + α-toco (400 µM). No significant differences were observed between the untreated (control) and vehicle-treated cells.

However, enhanced ELOVL1 activity could also be involved in the increased level of VLCFA. At the moment, seven enzymes termed ELOVL 1–7 (Elongation of Very-Long-Chain Fatty Acid), which are localized in the endoplasmic reticulum, have been identified. ELOVL1 is suggested to control VLCA synthesis up to C26:0. This is the most potent elongase for C24:0 and C26:0, whereas, depending on the cell type considered, similar elongase activity have been reported with ELOVL3 and ELOVL6 [94,95]. Our results also support an increase in the elongase activity index (which could correspond to ELOVL1, 3, and 6 activity; ratio (C24:0/C22:0), and ratio (C26:0/C22:0)) under treatment with 7β-OHC; these different elongase activity indexes were also strongly attenuated when 7β-OHC was associated with PLSO or α-tocopherol (Figure 7).

**Figure 8:**
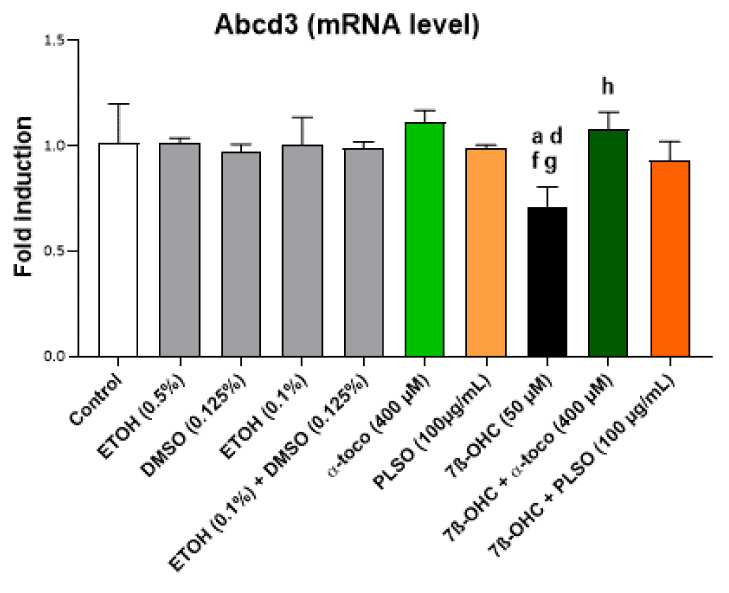
**Effects of 7β-hydroxycholesterol with and without *Pistacia lentiscus* L. on Abcd3 gene expression.** C2C12 cells were incubated for 24 h with or without 7β-OHC (50 µM) in the presence or absence of PLSO (100 µg/mL) or α-tocopherol (400 µM). The mRNA expression of Abcd3 was evaluated by qRT-PCR. Data shown are representative of three independent experiments. A multiple comparative analysis between the groups, taking into account the interactions, was carried out using an ANOVA test followed by a Tukey’s test. A *p*-value less than 0.05 was considered statistically significant. The statistically significant differences between the groups, which are indicated by different letters, take into account the vehicle used. a: comparison versus control; b: comparison versus ETOH (0.5%); c: comparison versus DMSO (0.125%); d: comparison versus ETOH (0.1%); e: comparison versus (ETOH (0.1%) + DMSO (0.125%)); f: comparison versus α-toco (400 µM); g: comparison versus PLSO (100 µg/mL); h: comparison versus 7β-OHC (50 µM); i: comparison versus 7β-OHC (50 µM) + α-toco (400 µM). No significant differences were observed between the untreated (control) and vehicle-treated cells.

In addition, as shown by qRT-PCR, the important decreases in the Abcd3 mRNA levels, observed under treatment with 7β-OHC (50 µM), were prevented by treatment with PLSO (100 µg/mL), as well as α-tocopherol (400 µM) (Figure 8).

### 3.8. Evaluation by Transmission Electron Microscopy of the Effects of Pistacia lentiscus *L*. Seed Oil on 7β-Hydroxycholesterol-Induced Cellular, Mitochondrial, and Peroxisomal Changes

**Figure 9:**
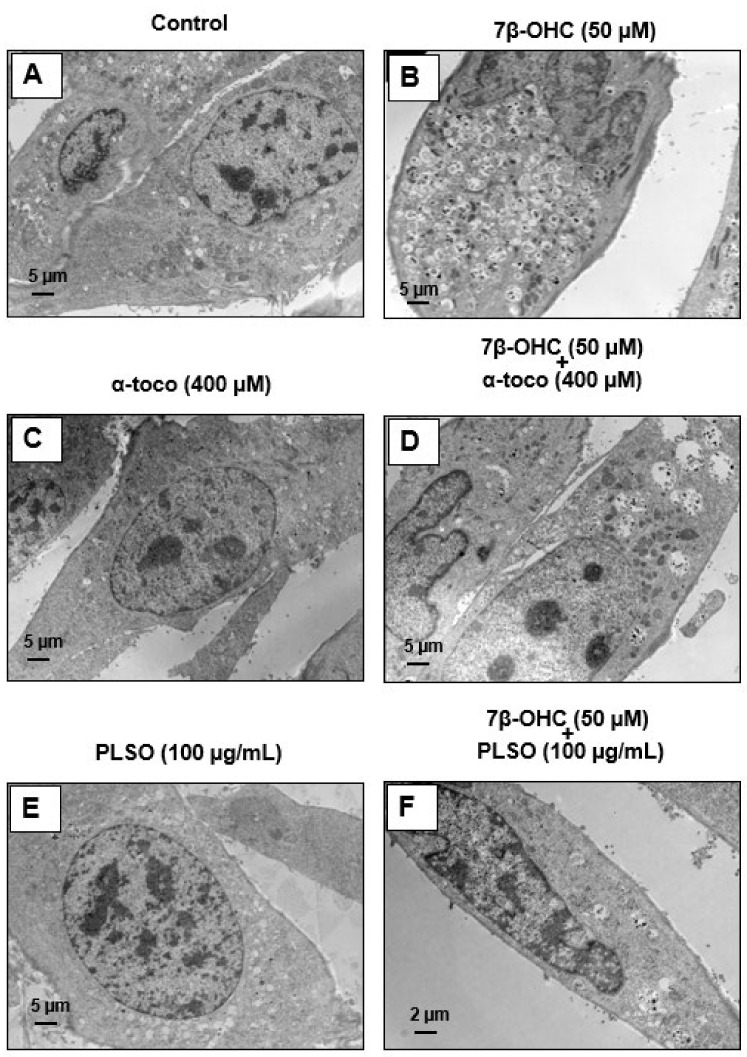
**Analysis of morphological changes in C2C12 myoblasts using transmission electron microscopy.** C2C12 cells were incubated for 24 h with or without 7β-OHC (50 µM) in the presence or absence of PLSO (100 µg/mL) or α-tocopherol (400 µM). In untreated cells (control) (**A**), α-tocopherol (400 µM)-treated cells (**C**), and PLSO (100 µg/mL)-treated cells (**E**), cells have a fusiform shape, with large round central nuclei containing several nucleoli; they have several small empty vacuoles and morphologically normal mitochondria and peroxisomes. In the 7β-OHC (50 µM)-treated cells (**B**), cells have an abnormal morphology: they have a round shape, irregular nuclei, and a lot of cytoplasmic vacuoles containing cell debris, as well as altered mitochondria and peroxisomes. In (7β-OHC + α-tocopherol and 7β-OHC+ PLSO)-treated cells, mainly morphologically normal cells were observed; they contain empty vacuoles and have mainly mitochondria and peroxisomes resembling those present in the control cells (**D–F**). No differences were observed between the control (**A**), α-tocopherol-treated (**C**), and PLSO-treated cells (**E**). Representative TEM images of the C2C12 myoblasts cultured in the presence of the vehicles are shown in Supplementary Figure S10.

**Figure 10:**
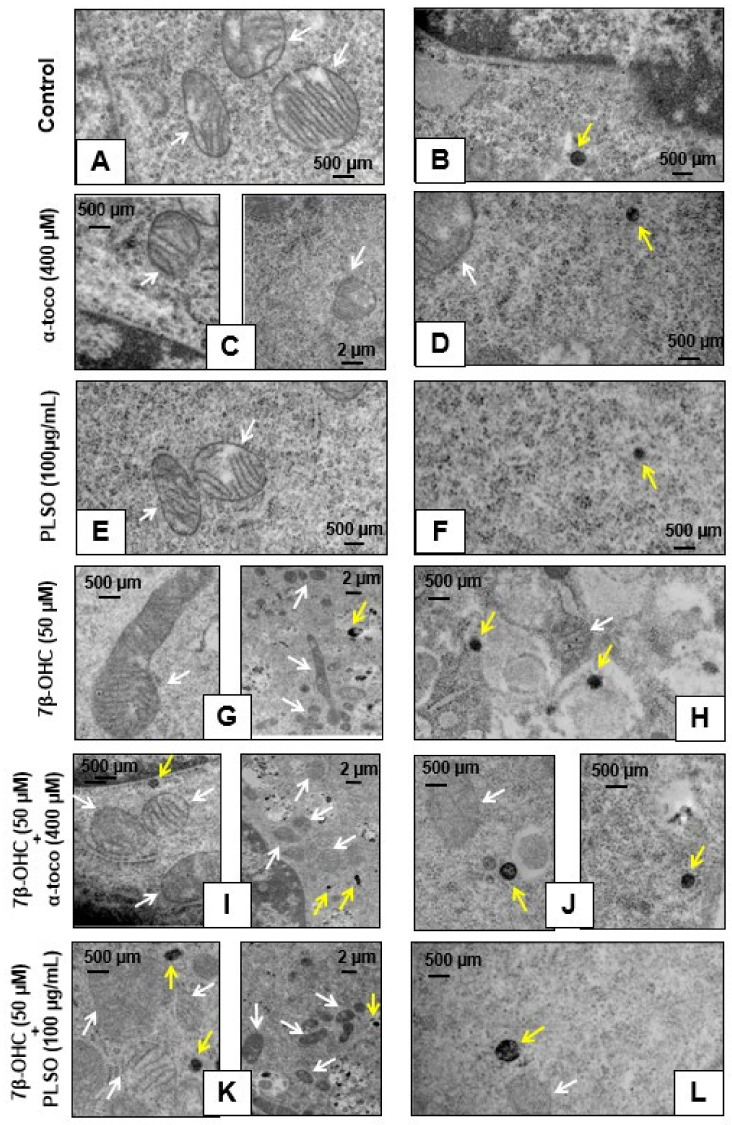
**Visualization of the mitochondria and peroxisomes in C2C12 myoblasts by transmission electron microscopy.** C2C12 cells were incubated for 24 h with or without 7β-OHC (50 µM) in the presence or absence of PLSO (100 µg/mL) or α-tocopherol (400 µM). In untreated cells (control) (**A**,**B**), α-tocopherol (400 mM)-treated cells (**C**,**D**), and PLSO (100 µg/mL)-treated cells (**E**,**F**), numerous mitochondria with clear cristae as well as round and regular peroxisomes were detected. In 7β-OHC (50 µM)-treated cells (**G**,**H**), irregular mitochondria with an increased size, reduced matrix density, and disrupted cristae, as well as peroxisomes with abnormal sizes and shapes were visualized. In (7β-OHC + α-tocopherol)-treated (**I**,**J**) and (7β-OHC+ PLSO) (**K**,**L**)-treated cells, mainly mitochondria and peroxisomes morphologically similar than those present in the control cells were observed. The white arrows point towards mitochondria and the yellow arrows point towards peroxisomes.

Transmission electron microscopy analysis was realized to study the morphological changes in C2C12 myoblasts (Figure 9 and Figure 10).

Control cells (Figure 9A), α-tocopherol (400 mM)-treated cells (Figure 9C), and PLSO (100 µg/mL)-treated cells (Figure 9E) have a well-preserved cell morphology with a fusiform shape and a large central round nucleus containing some nucleoli; in the cytoplasm, they have small empty vacuoles and morphologically normal mitochondria and peroxisomes. Compared to C2C12 control cells, 7β-OHC (50 µM)-treated cells showed significant alterations in cell morphology: most often round cells with irregular nuclei were observed, they contained several cytoplasmic vacuoles associated with a lot of cell debris as well as altered mitochondria and peroxisomes (Figure 9B). This disturbed morphology was attenuated by α-tocopherol (400 mM) and PLSO (100 µg/mL) treatment (Figure 9D,F). α-Tocopherol- and PLSO-treated cells (Figure 9C,E) have a similar morphology than control cells (Figure 9A). No morphological differences were observed between the control (Supplementary Figure S10A) and vehicle-treated cells (Supplementary Figures S10B,D).

Moreover, TEM observation of the C2C12 cells allowed us to highlight the essential cellular constituents, namely, mitochondria and peroxisomes. Control cells, α-tocopherol (400 mM)-treated cells, and PLSO (100 µg/mL)-treated cells have morphologically normal mitochondria with numerous cristae as well as round peroxisomes that are homogeneous in size in the range of 0.4 ± 0.1 µm (Figure 10A,F). However, major changes in the size and shape of these organelles were observed when C2C12 cells were treated with 7β-OHC; thus, several mitochondria with abnormal sizes and shapes were observed: larger size, reduced matrix density, and disrupted cristae (Figure 10G). In addition, several peroxisomes were detected in numerous cytoplasmic vacuoles, evocating a pexophagy process (Figure 10G,H). It is noteworthy that these changes in mitochondrial and peroxisomal topography and/or morphology were attenuated when 7β-OHC was combined with α-tocopherol (400 µM) or PLSO (100 µg/mL) (Figure 10I,L).

Indeed, we note that the mitochondria returned to their rounded shapes and the peroxisomes present in the vacuoles were rarely detected. Altogether, our data by TEM confirm that 7β-OHC induced several mitochondrial and peroxisomal changes, and that α-tocopherol and PLSO have strong cytoprotective effects on these organelles.

## 4. Discussion

**Figure 11:**
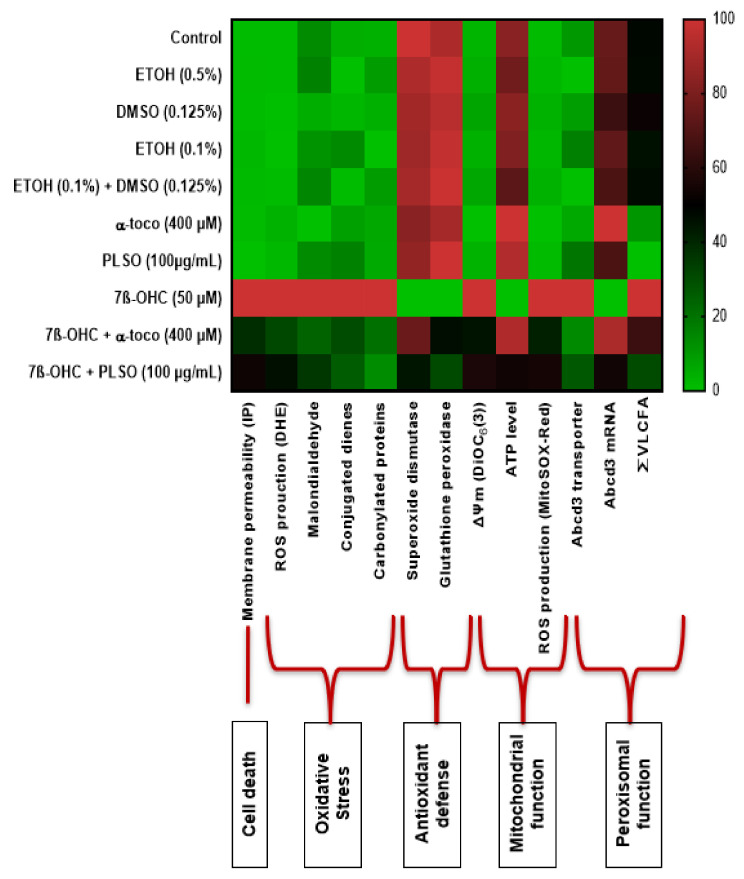
Heatmap representation of the toxicity of 7β-hydroxycholesterol and of the cytoprotective effects of PLSO and α-tocopherol on C2C12 cells. Heatmap graded from green (little or no effect: 0) to red (maximum effect: 100).

Aging is characterized by the variable decline in many biological functions, which can seriously alter the life quality of elderly people. Among the major alterations occurring during the aging process is sarcopenia, which corresponds to a loss of mass, quality, and strength of skeletal muscles [2,3]. Sarcopenia is generally accompanied by an impairment in muscle regeneration and a rupture of RedOx homeostasis, leading to ROS overproduction, which may, in turn, lead to the loss of muscle function [96]. ROS overproduction can favor lipid peroxidation, and increased levels of cholesterol auto-oxidation products, such as 7KC and 7β-OHC, are known to contribute to the development of several age-related diseases [31,81]. Interestingly, low physiological levels of ROS help maintain and heal skeletal muscle [97]; yet, tissue repair delay is caused by an excessive amount of ROS in the muscles, resulting in worsening the injury and creating atrophy [98]. Among the factors known to increase antioxidant defense and protect muscle from harmful effects of oxidative stress is nutrition. In that regard, in the current study, PLSO has been shown to contain a lot of compounds with antioxidant properties and this edible Mediterranean oil has a protective and antioxidant activity against 7β-OHC-induced cytotoxicity in C2C12 skeletal muscle cells. The data obtained are summarized in a heatmap (Figure 11).

Plants are an important source of bioactive molecules with therapeutic potential [99]. The genus *Pistacia* is a particular genus of the Anacardiaceae family due to its dioeciousness and naked flowers [100]. The species *Pistacia lentiscus* L. is a medicinal plant that grow wild in forests, low mountains, and in all types of soil [101]. Despite its limited distribution in the world, *Pistacia lentiscus* L. is known worldwide for several therapeutic properties, such as antioxidant, anti-inflammatory, anti-proliferative, and neuro-protective effects [48,49,50,51].

Using C2C12 cells cultured in the presence of 7β-OHC associated with many age-related diseases [31], our results show why there is interest in PLSO to prevent skeletal muscle cell dysfunction in a pro-oxidant environment. The results obtained establish that PLSO strongly attenuates the toxicity of 7β-OHC against which few cytoprotective molecules or mixtures of molecules have been identified [28]. Noteworthy, PLSO, which has a high nutritional value based on its biochemical profile established in this study, has a cytoprotective effects against 7β-OHC, which is of the same order of magnitude as that observed with α-tocopherol used at high concentration.

In the present study, we report that PLSO from Tunisia (area of Tabarka) has comparable amounts of total phenolics than PLSO from Algeria and Morocco (28.50 ± 0.77 mg/GAE/g vs. 25.15 ± 1.01 mg/GAE/g and 22.61 ± 1.42 mg/GAE/g, respectively) [102,103]. This similarity may be due to the fact that these three regions are in the same bioclimatic area. However, the flavonoids content of the PLSO used in this study was higher than the PLSO from Morocco [103].

The PLSO from Tunisia also showed a notable quantity of carotenoids. Thus, we could consider that PLSO is a great natural source for these pigments when compared to virgin olive oil (1.58–2.84 mg/kg of oil) [53]. These pigments, mainly β-carotene, are precursors of vitamin A. Dietary carotenoids are antioxidants thought to provide health benefits in the prevention of cardiovascular diseases and cancer [104,105]. In addition, β-carotene given to 8-week-old male mice by oral gavage for 7 or 14 days was able to maintain and enhance skeletal muscle mass by increasing the expression level of insulin-like growth factor-1 (IGF-1) [106]. In addition, fatty acids are considered a genetic code of oils; they are major components of most naturally occurring lipids in plants. The analysis of the fatty acid profile of PLSO from Tunisia is in accordance with the finding of Brahmi et al., (fatty acid profile expressed in g/100 g of PLSO) [102] and Dhifi et al., (fatty acid profile expressed in %) [107]. It has been reported that the most abundant unsaturated fatty acids present in PLSO were oleic acid (OA; C18:1 n−9) and linoleic acid (LA; C18:2 n−6). OA is reputed for its effect on oils oxidative stability and its nutritional value [108]. OA also has strong antioxidant activities against 7-KC-induced cell death on murine microglial BV-2 cells [55,109]. LA is also an essential fatty acid and a precursor of polyunsaturated fatty acids with longer chains, which enhances the nutritional value of the vegetable shortening [110]. Lee et al., (2009) indicated that unsaturated fatty acids, especially OA and LA, enhanced the proliferation of C2C12 skeletal muscle cells [111]. In the current study, palmitic acid (C16:0) was the predominant saturated fatty acid found in PLSO, which is consistent with previous studies [107,112]. According to the literature, PLSO presents a higher palmitic acid content than olive oil (9.85–20.30%) and other vegetable oils, such as milk thistle seed oil (6.25–13.06%) and argan oil (12.11–13.05%) [62]. This saturated fatty acid has been thought to increase the total cholesterol, and specifically the LDL cholesterol levels, although a previous study demonstrated that high consumption of palmitic acid in healthy volunteers does not increase the cholesterol if it is combined with LA, as is the case in PLSO [113]. Noteworthy, the low saturated/unsaturated fatty acids ratio (0.404%) indicates that PLSO contains a huge amount of unsaturated fatty acids, which gives it valuable nutritional and dietetic value as well as curative properties [107].

The PLSO sterol profile also showed that β-sitosterol is the most abundant phytosterol (67.25 ± 3.24 mg/100 g of oil) followed by α-epoxysitostanol and 24-methylene cycloartenol. β-sitosterol was also the most representative sterol in the PLSO harvested from different Tunisian locations but the amount of this sterol changed according to geographic origin (99.61 mg/100 g of oil in Korbousand; 389.50 mg/100 g of oil in Rimel) [52]. A lower amount of β-sitosterol was found in Algerian PLSO (58.79 ± 1.19 mg/100 g of oil) [102]. β-sitosterol is one of the most abundant dietary phytosterols that have potential health benefits. Several experimental studies demonstrated that β-sitosterol could regulate the glucose and lipid metabolism [114] and inhibit inflammation and oxidative stress [115,116]. In addition, an in vitro study showed that C2C12 skeletal muscle cells treatment by β-sitosterol improves mitochondrial biogenesis and function via increasing mitochondrial electron transport and energy demand and by activating protein kinase/PGC-1 [117]; these observations give the PLSO a great nutritional and therapeutic value.

In addition, tocopherols are also major ingredients in the oils since they have high antioxidant activity [118]. They could protect polyunsaturated fatty acids (PUFA) from oxidation by scavenging lipid peroxyl radicals (ROO^•^) [119]. In PLSO, α-tocopherol is present in the highest quantity and contributes to the natural conservation of PLSO. It is important to highlight that PLSO is an excellent source of vitamin E, which is constituted of four tocopherols and four tocotrienols [120].

In another hand, the antioxidant potential of PLSO, evaluated by DPPH, FRAP, and FIC assays, demonstrated an important antioxidant potential of this oil, reinforcing our interest to study the cytoprotective properties of this oil in vitro. Consequently, in the context of sarcopenia, we evaluated the protective properties of PLSO against 7β-OHC-induced cytotoxicity on a model of murine C2C12 myoblast cells.

Indeed, several oxysterols, including 7β-OHC, are present at increased levels and high amounts in the plasma and tissues of patients with age-related diseases [31,121], and our preliminary data obtained by GC-MS on the plasma from subjects with and without sarcopenia have revealed significant higher plasma levels of 7β-OHC in sarcopenic patients. It has been shown that 7β-OHC, which is a potent inducer of oxidative stress by stimulating at least in part NAD(P)H activity [122], was among the most cytotoxic oxysterol on different cell types from different species [42]. Oxidative damage is supposed to be the main responsible factor for cellular aging. A potential oxidative alteration of satellite cells could induce problems in muscle regeneration, as is the case in aging [123]. Therefore, in age-related diseases, including sarcopenia, the identification of molecules or mixture of molecules capable to attenuate 7β-OHC-induced cell death, defined as oxiapoptophagy [124], has a crucial interest to prevent and/or treat these diseases.

In the present study, 7β-OHC (50 µM, 24 h) showed cytotoxic effects on C2C12 myoblasts cells, which are characterized by an induction of cell death associated with ROS overproduction as well as mitochondrial and peroxisomal dysfunction. Some of these effects were previously obtained on vascular cells, hematopoietic and immune cells, retinal cells, and nerve cells exposed to 7β-OHC [30,109,125,126]. As previously reported on numerous adherent cells, 7β-OHC induces a loss of cell adhesion on C2C12 cells, which is characterized by an increase in round and floating cells, suggesting an alteration of membrane constituents associated with cell death. These alterations could be triggered by a RedOx imbalance and an induction of oxidative stress, which could modify the structure and the physical properties of plasma membranes, favoring the degradation of adhesion molecules and cell junctions by mechanisms involving the ROS-dependent activation of matrix metalloproteinases [127]. In addition, 7β-OHC-induced plasma membrane modifications, revealed in the present study by an increased permeability to PI, could modify the ionic homeostasis (Ca^2+^, N^+^, K^+^) with important consequences on numerous signaling pathways, especially those involved in the activation of apoptosis [30,128] or the transmission of nerve influx [129]. In sarcopenic patients, the alteration in nerve influx could also amplify muscle dysfunction at the neuro–muscular junction. In support of the key role of oxidative stress in 7β-OHC-induced cell death [124], our results obtained in C2C12 cells showed that 7β-OHC also induced an overproduction of mitochondrial ROS, associated with an accumulation of lipid and protein oxidation products, such as MDA, CDs, and CPs, as well as a decrease in the major antioxidant enzymes activities (superoxide dismutase (SOD) and glutathione peroxidase (GPx)). These results evocate the cytotoxic effects observed with 7KC on different types of neuronal cells (158 N murine oligodendrocytes, BV-2 murine microglial cells, and N2a murine neuronal cells) on which the activation of the oxidative stress is at the origin of the toxicity of this oxysterol [126,130].

On C2C12 cells as well as on other cell types, under treatment with 7β-OHC, it can be considered that ROS overproduction results from the activation of different NADPH oxidase isoforms [35] and from mitochondrial dysfunctions. As ROS overproduction and mitochondrial dysfunction are considered as major phenomena involved in senescence and aging [131], our data support the hypothesis that 7β-OHC could contribute to the aging process in skeletal muscle cells. 7β-OHC induces ROS overproduction probably also contributes to the alterations in mitochondrial structures and of mitochondrial proteins present in the mitochondrial complexes contributing to oxidative phosphorylation. This could favor not only a loss of transmembrane mitochondrial potential (Δψm) but also a disruption of the respiratory chain function and a limitation in energy production, leading to the decreased ATP production observed in the present study. Consequently, in tissues with a low cell turnover, such as the skeletal muscle, alteration of the mitochondria under the action of 7β-OHC may have important detrimental effects on the muscular function.

Like mitochondria, the peroxisomes, which are metabolically tightly connected to the mitochondria [132], represent another important source of intracellular ROS (mainly H_2_O_2_), and it is now well established that peroxisomal dysfunctions increase ROS overproduction and disturb mitochondrial activity [80,133]. It has been shown on 158 N murine oligodendrocytes that the inactivation of the peroxisomal transporters ABCD1 and 2 associated with peroxisomal β-oxidation, as well as of ACOX1, which is the main limiting enzyme of peroxisomal β-oxidation, favor oxidative stress and increase ROS production in whole cells and at the mitochondrial level [134]. The peroxisome, in addition to its implication in the regulation of RedOx homeostasis, is implicated in the control of lipid metabolism and non-cytokinic inflammation [135]. It has also been suggested that the peroxisome may also play a crucial role in cellular aging [136]. However, still little is known about the contribution of the peroxisome in the aging process but an involvement of this organelle in the amplification of mitochondrial dysfunction is quite well documented [93]. The present study realized on C2C12 cells clearly shows peroxisomal alterations in the presence of 7β-OHC, which is characterized by a reduced peroxisomal density and a lower level of Abcd3 peroxisomal transporter. As 7β-OHC could affect and reduce the peroxisomal transport and degradation of VLCFA (C24:0, C24:1, C26:0, and C26:1), whose intracellular accumulation can have toxic consequences [137], the levels of VLCFA have been measured by GC-MS and the elongase activity index of the enzyme ELOVL1 associated with VLCFA metabolism has been determined [138]. Measuring the level of some VLCFA in sarcopenia could be of interest since some fatty acids behave as metabolic inhibitors, uncouplers of oxidative phosphorylation, and membrane permeability transition (MPT) inducers; it is thus hypothesized that these pathophysiological mechanisms could contribute to the muscular symptoms in sarcopenia [139].

Based on the results obtained on C2C12, preventing the toxicity of 7β-OHC, which is essentially formed by auto-oxidation of cholesterol and is increased in many age-related diseases, remains a major challenge [28]. For this purpose, it is still necessary to identify the molecules or mixtures of molecules allowing to prevent or reduce its toxicity. Indeed, at the moment only few natural and synthetic molecules as well as mixtures of molecules capable to inhibit 7β-OHC-induced cytotoxicity have been identified [28]. Noteworthy, we reported that PLSO is an edible oil with high nutritional value containing several antioxidant nutrients known for their protective effects against various diseases associated with oxidative stress, and our data indicate that PLSO exhibits strong cytoprotective activities against 7β-OHC on C2C12 mouse skeletal muscle cells. The effects observed with PLSO were in the range of order of those obtained with α-tocopherol known to strongly counteract 7β-OHC-induced oxidative stress and cell death induction on several cell types [30]. In accordance with these findings, it has been reported that PLSO was able to inhibit H_2_O_2_-induced oxidative stress in human skin culture [140]. Besides PLSO, other oils and natural bioactive compounds, such as Schisandrae semen essential oil [141], isorhamnetin [142], resveratrol [143], and phloretin [144], were described as antioxidant molecules in C2C12 murine skeletal muscle cells. Our data clearly show that PLSO attenuates both mitochondrial and peroxisomal dysfunctions induced by 7β-OHC through the restoration of succinate dehydrogenase activity and Δψm, a reduction in mitochondrial ROS production, normalization of Abcd3 expression, and VLCFA levels. Thus, PLSO acts on the major targets involved in aging—those contributing to the development of major age-related diseases—namely, oxidative stress, mitochondria, and peroxisome. The cytoprotective results obtained with LPSO evocate those obtained with several other oils associated with the Mediterranean diet (olive oil, milk thistle seed oil, and argan oil) [86,109]. Thus, the value of the lipid mixtures is underscored by these different data to restrain cell death and oxidative stress induced by oxysterols.

## 5. Conclusions

This study demonstrates that 7β-OHC triggers oxidative stress, mitochondrial and peroxisomal dysfunction, and cell death on C2C12 myoblast cells. Noteworthy, in the presence of PLSO as well as of α-tocopherol, these different cytotoxic effects were strongly attenuated and PLSO was as efficient as α-tocopherol used at a high concentration. Noteworthy, as MitoQ, which selectively accumulates in the mitochondria, did not attenuate 7β-OHC-induced cell death, our data suggest that attenuation of mitochondrial dysfunction is not sufficient to counteract 7β-OHC-induced cell death, and that PLSO, which strongly reduces mitochondrial dysfunction, also act on other cellular targets. On the basis of the biochemical composition of PLSO (fatty acids, tocopherols, and polyphenols), of its antioxidant properties, and of its cytoprotective effects, it is suggested that a diet associated with this oil could contribute to the prevention of skeletal muscle dysfunctions. In a therapeutic context, the bioavailability and the efficiency of the biological compounds present in PLSO could be improved using a number of approaches. These later could include micro- and nano-encapsulation strategies, chimeric tractable molecules, and targeted-specific cell compartments and organelles (mitochondria, peroxisomes), such as Targeted Organelle Nano-therapy (TORN-therapy) [145,146] as well as functional foods.

## Figures and Tables

**Table 1:** Total phenols, flavonoids, and carotenoids contents of *Pistacia lentiscus* seed oil (PLSO).

	Total Phenols (mg GAE/g of Extract)	Flavonoids (mg CE/g of Extract)	Carotenoids (mg/kg)
***Pistacia lentiscus* seed oil**	28.50 ± 0.77	51.36 ± 2.30	2083.59 ± 55.00

GAE: gallic acid equivalent; CE: catechin equivalent. Each value represents the mean of three determinations ± standard deviation.

**Table 2:** *Pistacia lentiscus* seed oil polyphenol content (mg equivalents quercetin/100 g of oil).

Polyphenols	(mg Equivalents Quercetin/100 g of Oil)
**Protocatechuic acid**	0.140 ± 0.001
**Coumarin**	0.650 ± 0.003

Polyphenols chromatogram obtained by HPLC (Supplementary Figure S2). Each value represents the mean of three determinations ± standard deviation.

**Table 3:** *Pistacia lentiscus* seed oil fatty acids profile (g/100 g of oil; % of total fatty acids).

Fatty Acids	g/100 g of Oil (*)	% (**)
***∑SFA***	32.333 ± 0.566	28.77 ± 0.24
Myristic acid (C14:0)	0.015 ± 0.000	0.05 ± 0.01
Palmitic acid (C16:0)	30.457 ± 0.355	27.20 ± 0.22
Margaric acid (C17:0)	0.022 ± 0.003	0.05 ± 0.00
Stearic acid (C18:0)	1.527 ± 0.124	1.26 ± 0.01
Arachidic acid (C20:0)	0.041 ± 0.012	0.12 ± 0.00
Behenic acid (C22:0)	0.271 ± 0.072	0.04 ± 0.00
Lignoceric acid (C24:0)	ND	0.05 ± 0.00
***∑UFA***	94.706 ± 2.910	71.25 ± 0.28
***∑MUFA***	64.736 ± 1.827	53.67 ± 0.17
Palmitoleic acid (C16:1 n−7)	2.623 ± 0.084	2.25 ± 0.01
Heptadecenoic acid (C17:1)	0.179 ± 0.058	ND
Oleic acid (C18:1 n−9)	60.183 ± 1.556	49.77 ± 0.12
Vaccenic acid (C18:1 n−7)	1.670 ± 0.073	1.51 ± 0.03
Gadoleic acid (C20:1 n−9)	0.081 ± 0.056	0.14 ± 0.01
***∑PUFA***	29.970 ± 1.083	17.58 ± 0.11
Linoleic acid (C18:2 n−6)	29.665 ± 0.990	17.19 ± 0.10
α-linolenic acid (C18:3 n−3)	0.305 ± 0.093	0.39 ± 0.01
***∑SFA/∑UFA***	0.34 ± 0.19	0.41 ± 0.86

SFA: saturated fatty acids; MUFA: monounsaturated fatty acids; PUFA: polyunsaturated fatty acids; ND: not detected. Each value represents the mean of three determinations ± standard deviation. (*): data obtained at the University of Monastir (Monastir, Tunisia) with (C19:0) used as internal standard; (**) data obtained at INRAE (Dijon, France) without an internal standard. Data were obtained with the same sample of PLSO. The corresponding chromatograms are shown in Supplementary Figure S3A,B.

**Table 4:** *Pistacia lentiscus* seed oil phytosterol profile (mg/100 g of oil).

Phytosterol	(mg/100 g of Oil)
Campesterol	4.48 ± 0.85
Stigmasterol	2.69 ± 0.23
β-Sitosterol	67.25 ± 3.24
∆^5^-Avenasterol	3.10 ± 0.90
β-Amyrine	1.81 ± 0.19
Cycloartenol	9.35 ± 1.49
24-Methylene cycloartenol	16.10 ± 2.72
α-Epoxysitostanol	33.36 ± 1.65
Other phytosterols	16.76 ± 2.83
Total	154.89 ± 5.40

The phytosterol chromatogram obtained by GC-FID is shown in Supplementary Figure S4. Each value represents the mean of three determinations ± standard deviation.

**Table 5:** α-Tocopherol content (mg/kg) of *Pistacia lentiscus* seed oil (PLSO).

	α-Tocopherol (mg/kg)
***Pistacia lentiscus*** **seed oil**	68.10 ± 3.41

Values are the mean ± SD of three determinations.

**Table 6:** Antioxidant activity of *Pistacia lentiscus* seed oil (PLSO).

IC50 Values (mg/mL)				(Trolox Equivalent)
Samples	DPPH	FRAP	Iron Chelating (FIC)	KRL
**PLSO**	5.010 ± 0.095	1.15 ± 0.23	5.61 ± 0.14	4440.00 ± 493.60
**AO**	−	−	−	360.80 ± 153.60
**EDTA (standard)**	−	−	0.60 ± 0.09	−
**AA (standard)**	0.810 ± 0.270	0.41 ± 0.16	−	−
**α-tocopherol**	−	−	−	0.94 ± 0.01

Each value represents the mean of three determinations ± standard deviation. IC50: half-maximal inhibitory concentration; PLSO: *Pistacia lentiscus* L. seed oil; AO: argan oil, EDTA: ethylenediaminetetraacetic acid; AA: ascorbic acid; DPPH: 2,2-diphenyl-1-picrylhydrazyl; FIC: Ferrous Iron Chelating FRAP: Ferric Reducing Antioxidant Power; KRL: Kit Radicaux Libres. For the KRL test, data are presented in Trolox equivalent: 1 mL of PLSO is equivalent to X moles of Trolox (value shown in the table).

## Data Availability

Data from the in vitro study are available in the laboratory notebooks and on the computers from the Université de Bourgogne (EA7270) (Dijon, France) and from the University of Monastir (Monastir and Sousse, Tunisia). Data related to biochemical analysis and electron microscopy are stored on the computers from the Université de Bourgogne, INRAE (Dijon, France) and Lara-Spiral (Couternon, France).

## Supplementary Materials

The following are available online at https://www.mdpi.com/article/10.3390/antiox10111772/s1. Supplementary Figure S1: Different stages of preparation of the Pistachia lentiscus L. seed oil (PLSO) with seeds collected in Tunisia (area of Tabarka); Supplementary Figure S2: polyphenols chromatogram obtained by HPLC (protocatechuic acid and coumarin were identified based on their UV spectra (280 nm)); Supplementary Figure S3: Fatty acid chromatogram obtained by GC; Supplementary Figure S4: Phytosterol chromatogram obtained by GC-FID; Supplementary Figure S5: Plasma levels of cholesterol, 7β-hydroxycholesterol (7β-OHC) and 7-ketocholesterol in sarcopenic patients and non-sarcopenic patients; Supplementary Figure S6: Evaluation with the MTT assay of the effects of Pistacia lentiscus L. seed oil (PLSO) and 7β-hydroxycholesterol on C2C12 cell viability; Supplementary Figure S7A: Effect of 7β-hydroxycholesterol and Pistacia lentiscus L. seed oil (PLSO) on C2C12 cell morphology; Supplementary Figure S7B: Absence of effect of vehicles (EtOH 0.5%; DMSO 1.125%; EtOH 0.1%; EtOH 0.1%+ DMSO 0.125%) on C2C12 cell morphology; Supplementary Figure S8: Evaluation with the fluorescein diacetate (FDA) assay of the effect of MitoQ on 7β-hydroxycholesterol-induced cell death; Supplementary Figure S9: Biochemical pathway of the peroxisomal β-oxidation of very-long-chain fatty acids; Supplementary Figure S10: Comparison by transmission electron microscopy of cell morphology in control C2C12 myoblasts and vehicles-treated C2C12 myoblasts. Supplementary Table S1: List of available polyphenols spectra present in the database of LARA-Spiral company (Couternon, France).
