# Supplementary material for: Antioxidant Properties and Cytoprotective Effect of Pistacia lentiscus L. Seed Oil against 7β-Hydroxycholesterol-Induced Toxicity in C2C12 Myoblasts: Reduction in Oxidative Stress, Mitochondrial and Peroxisomal Dysfunctions and Attenuation of Cell Death
Source: Antioxidants (Basel). 2021 Nov 5;10(11):1772. doi: 10.3390/antiox10111772 (PMC8615043; doi:10.3390/antiox10111772)
Supplement: Supplementary file 1 [file antioxidants-10-01772-s001.zip › antioxidants-1420181-supplementary.pdf]

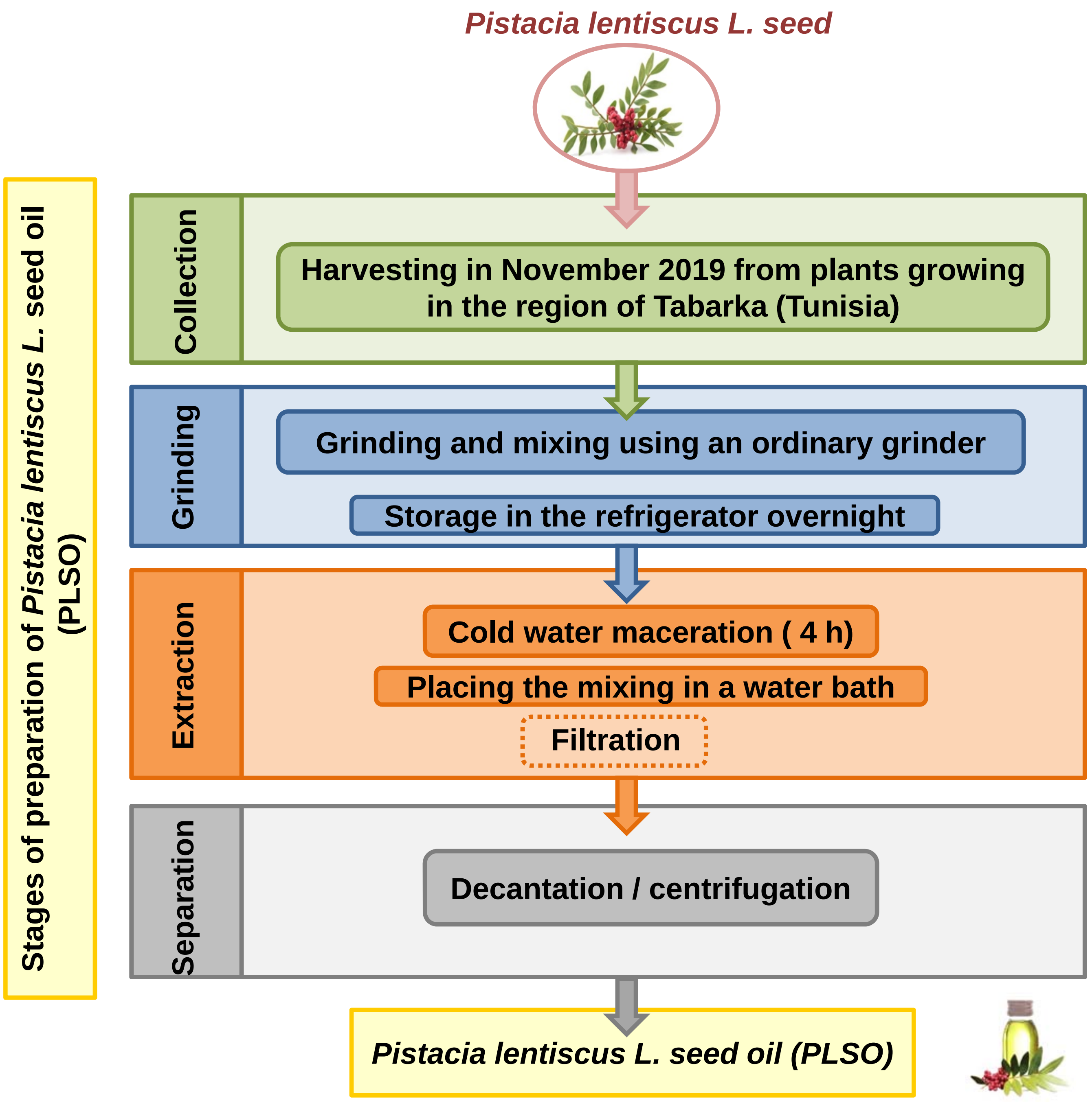

Supplementary Figure S1: Different stages of preparation of the *Pistachia lentiscus* L. seed oil (PLSO) with seeds collected in Tunisia (area of Tabarka).

Laboratoires spiral s.a.
LABORATOIRE DE RECHERCHES APPLIQUEES

C2104002RL - Gérard LIZARD - Polyphenols in PLSO.
Data obtained by Emmanuelle CAMUS-PROST; 2021.

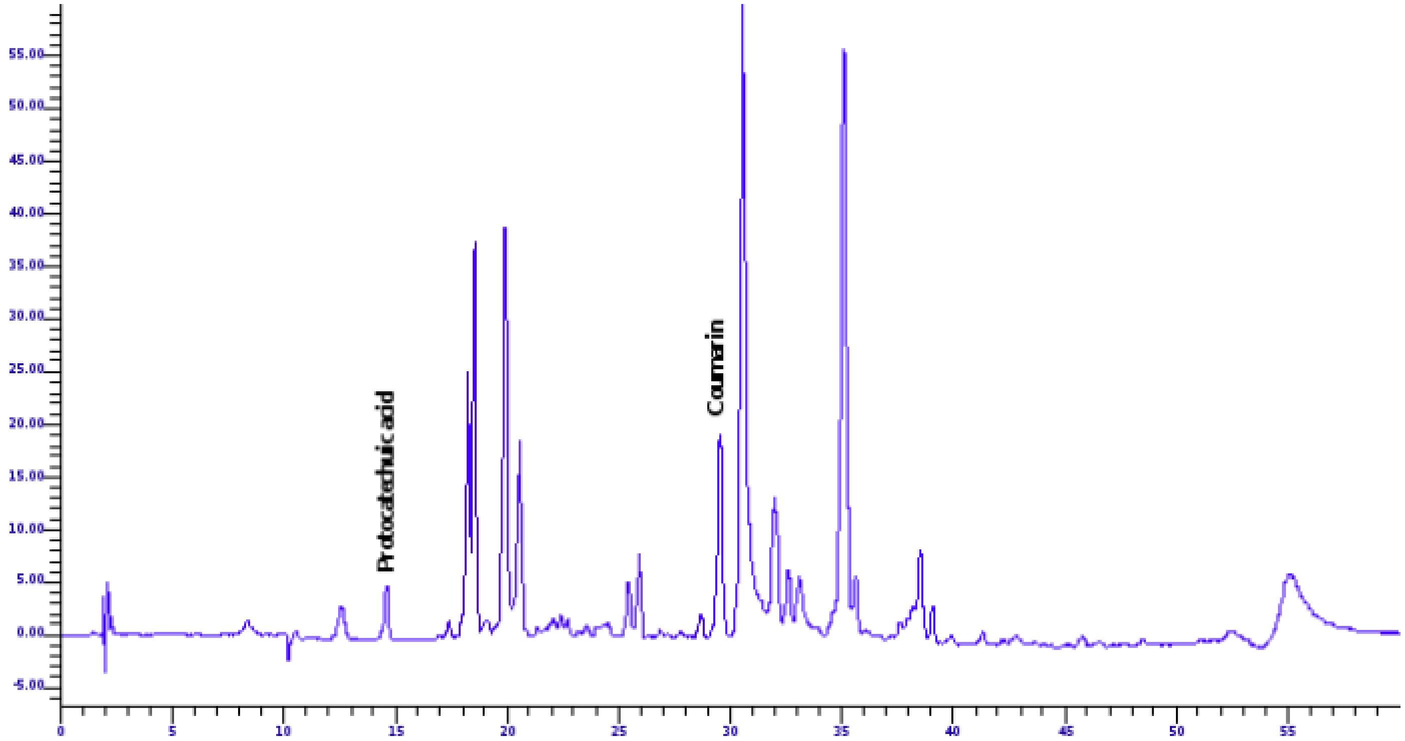

Supplementary Figure S2: polyphenols chromatogram obtained by HPLC (protocatechuic acid and coumarin were identified based on their UV spectra (280 nm)). The other peaks were not characterized.

- Data obtained by **Imed Cheraief**
HP 5890 Series II Gas Chromatograph,
(Hewlett Packard, San José, CA, USA)

- Data obtained by **Lucy Martine and Niyazi Acar**
Thermo Fisher Scientific Trace 1310 Gas Chromatograph
(Thermo Fisher Scientific, Waltham, MA, USA)

A

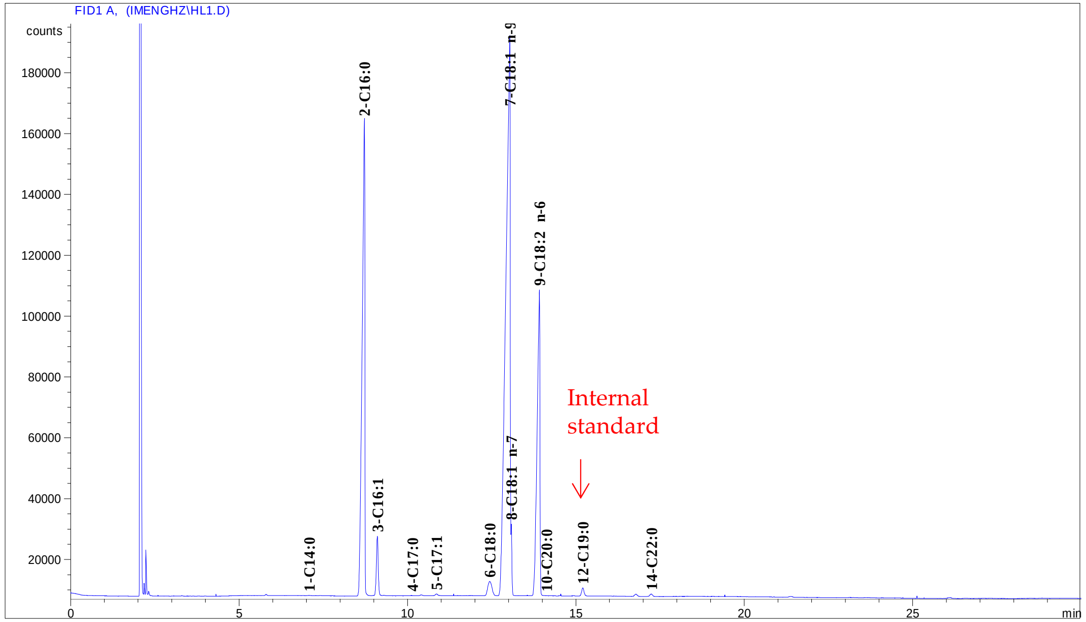

B

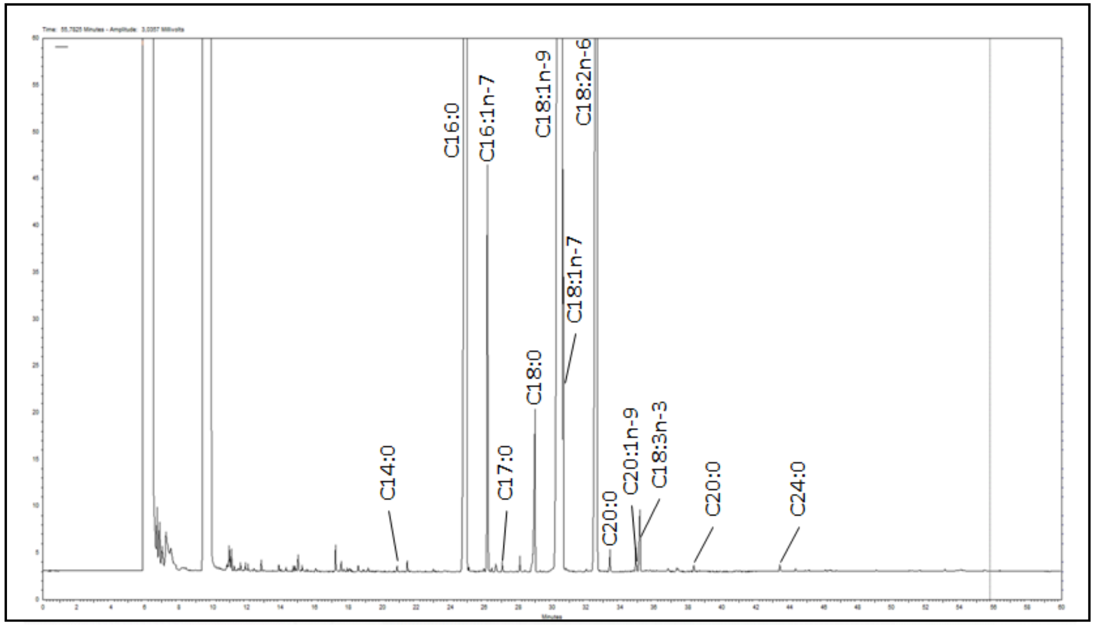

Supplementary Figure S3: Fatty acid chromatograms obtained by GC. (**A**) Chromatogram obtained at the University of Monastir (Monastir, Tunisia) with (C19:0) used as internal standard;

(**B**) chromatogram obtained at INRAE (Dijon, France) without internal standard.

- Data obtained by **Lucy Martine and Niyazi Acar**
HP 5890D Gas Chromatograph
(Hewlett Packard, San José, USA)

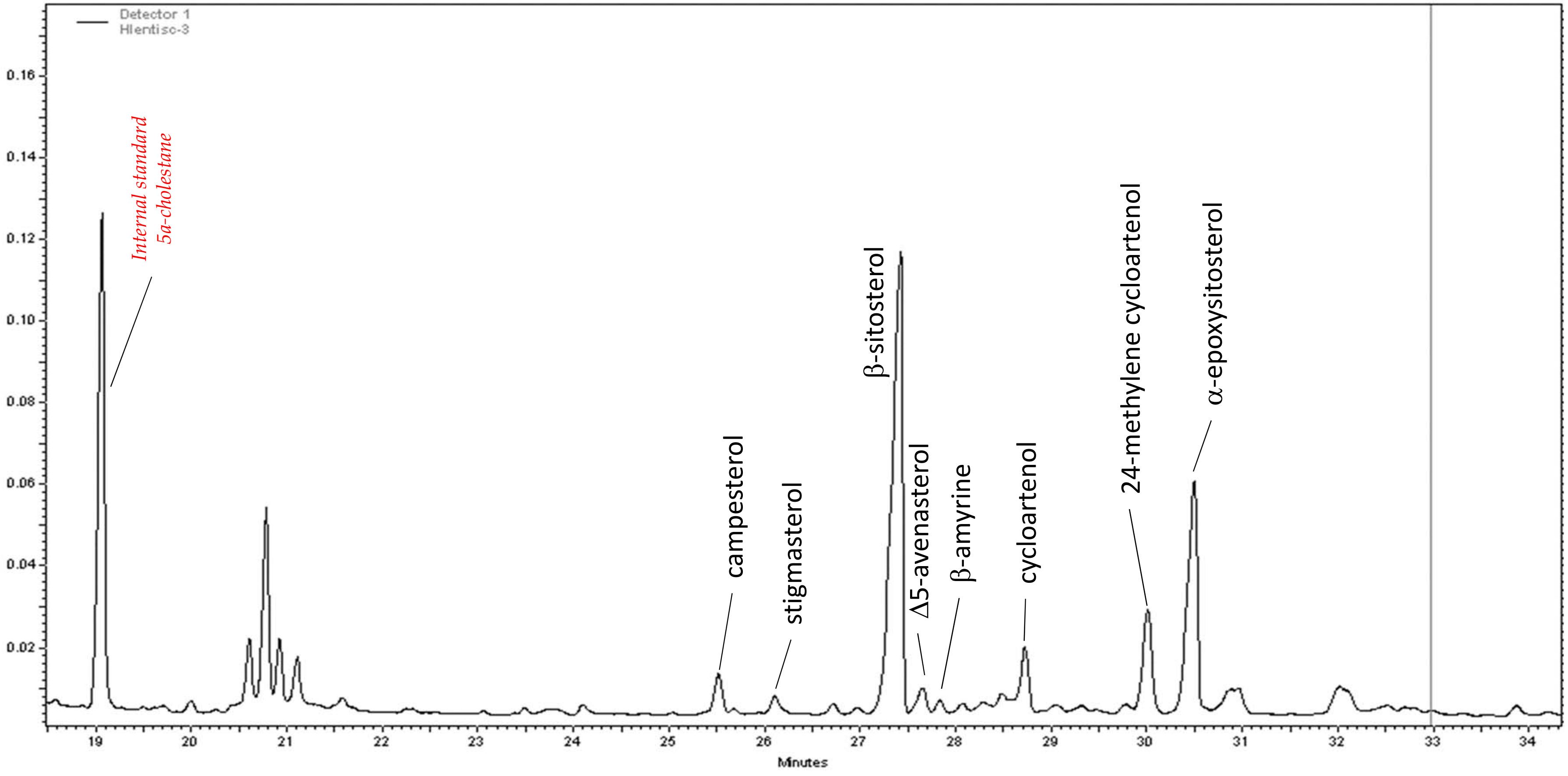

Supplementary Figure S4: Phytosterols chromatogram obtained by GC-FID.

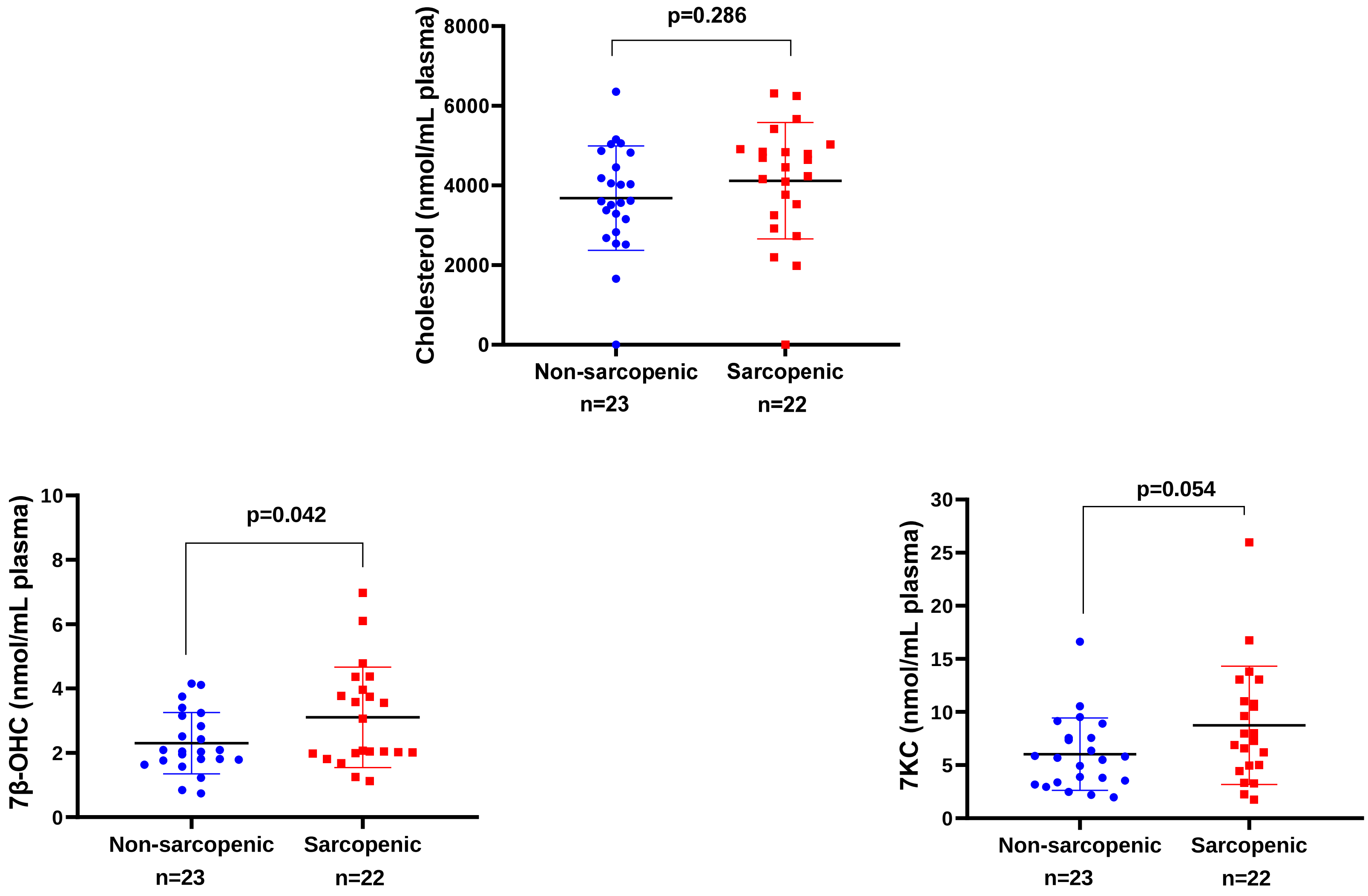

Supplementary Figure S5: Plasma levels of cholesterol, 7β-hydroxycholesterol and 7-ketocholesterol in sarcopenic patients and non-sarcopenic patients. 45 adults from 65 years old and more (23 men, 22 women) were recruited over a period of 1 month from January to February 2019. All participants were recruited from a nursing home (Sousse, Tunisia). The Test Timed Up and Go (TUG) was used to classify patients as sarcopenic (22 subjects; age= 80 ± 4.16; female/male= 15/7), and non-sarcopenic (23 subjects; age= 70.84 ± 4.38; female/male= 7/16). Oxysterols as well as cholesterol were measured by GC-MS in the plasma of non-sarcopenic and sarcopenic subjects. In sarcopenic patients, 7β-OHC level was significantly higher than in non-sarcopenic subjects whereas no significant difference in 7KC and cholesterol level was observed.

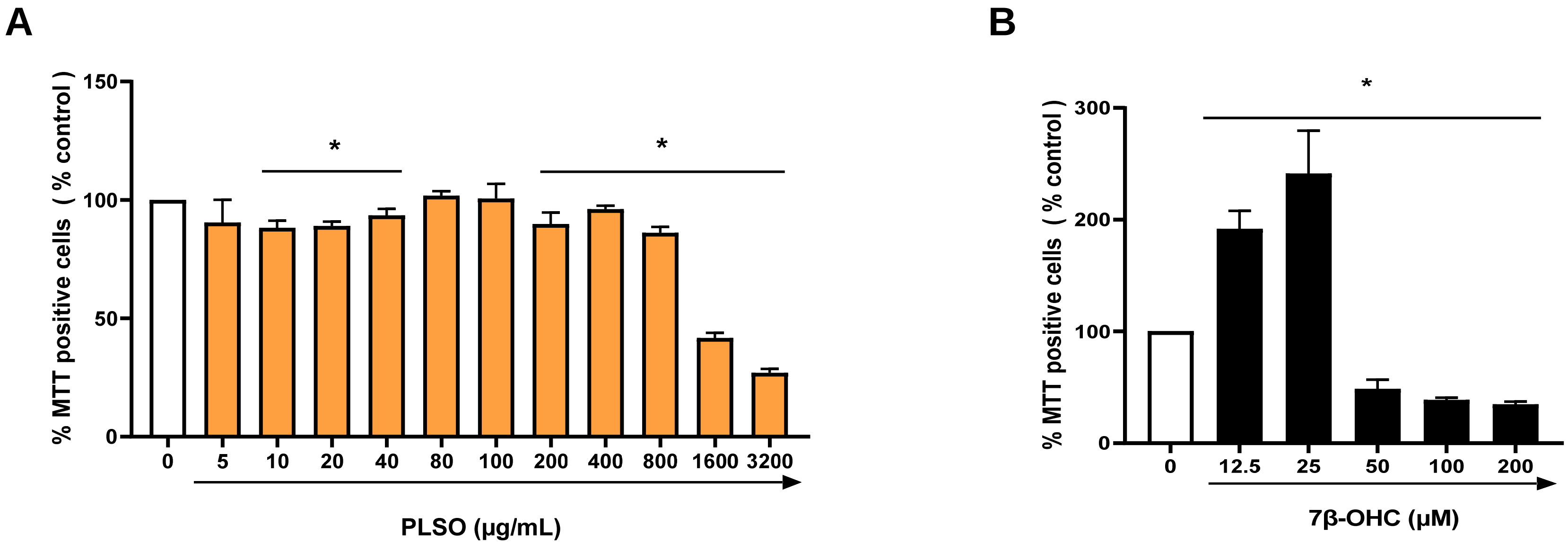

Supplementary Figure S6: Evaluation with the MTT assay of the effects of *Pistacia lentiscus* L. seed oil (PLSO) and 7β-hydroxycholesterol on C2C12 cell viability. A: C2C12 cells were incubated with PLSO at different concentrations (5-3,200 µg/mL; 24 h).
B: C2C12 cells were incubated with different concentrations of 7β-OHC (12.5 - 200 µM; 24 h). Cell viability was determined with the MTT assay,
Data are presented as percentage of untreated cells (control).
Data are expressed as mean ± SD of two independent experiments performed in triplicate. The assays were compared to control using a Student's t-test (*$p \leq 0.05$).

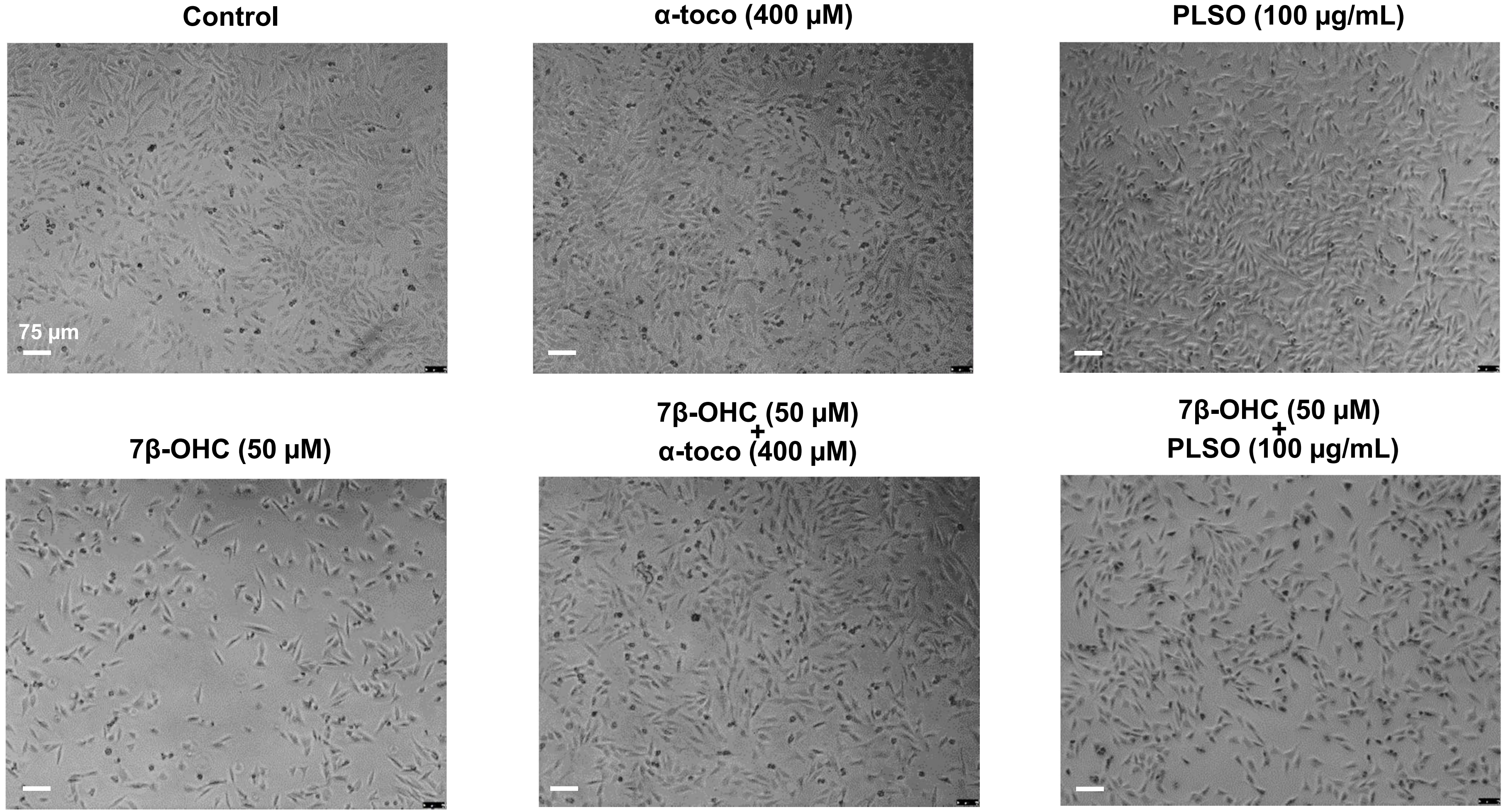

Supplementary Figure S7 A: Effect of 7β-hydroxycholesterol and *Pistacia lentiscus* L. seed oil (PLSO) on C2C12 cell morphology. C2C12 cells were incubated for 24 h with or without 7β-OHC (50 µM) in the presence or absence of PLSO (100 µg/mL) or α-tocopherol (400 µM). The protective effect of PLSO or α-tocopherol against 7β-OHC- induced modification of cell morphology was realized by phase-contrast microscopy. Further, phase-contrast images of C2C12 in vehicles are shown **in Supplementary Figure 4 B.**

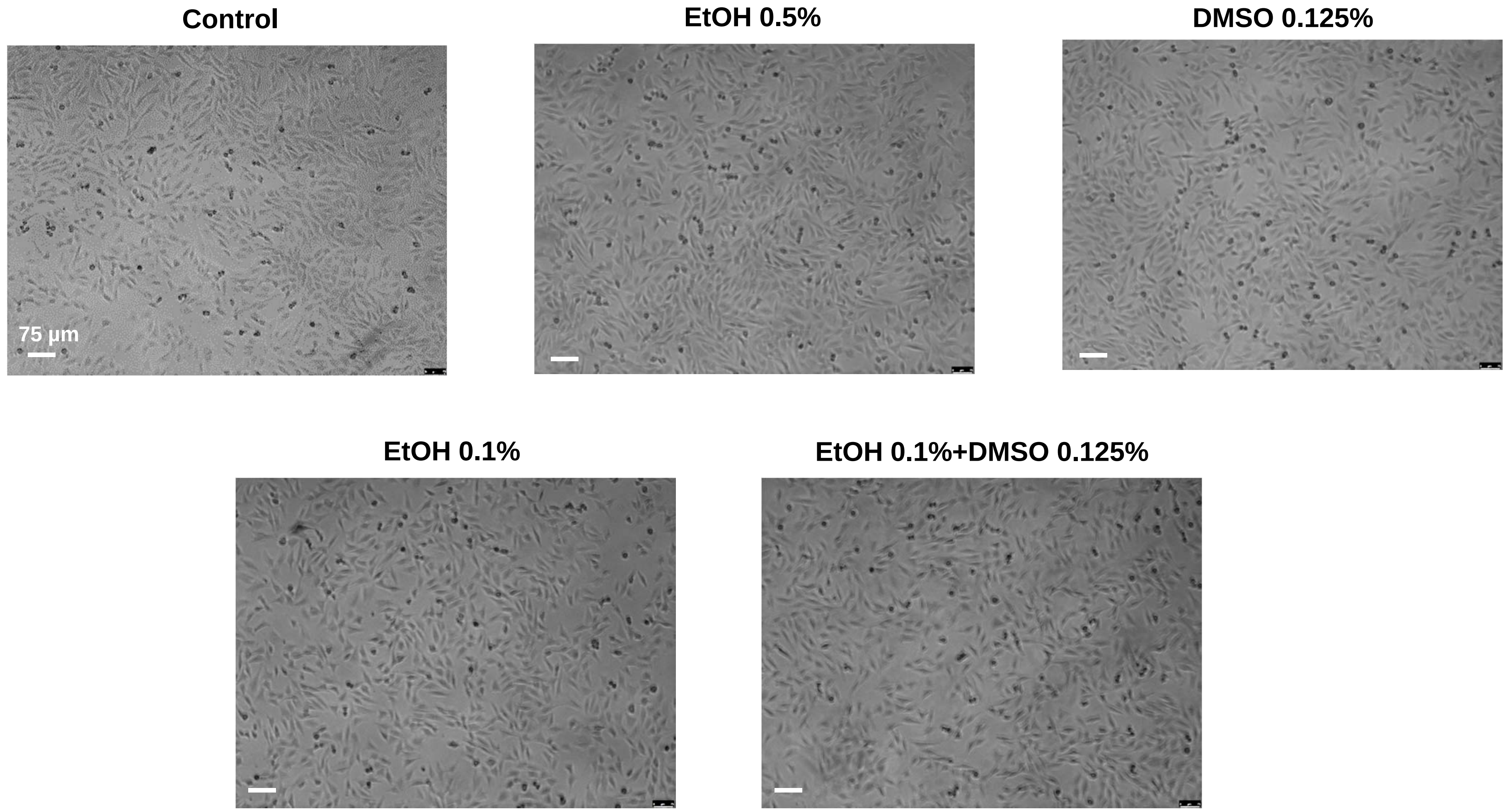

Supplementary Figure S7 B: Absence of effect of vehicles (EtOH 0.5%; DMSO 1.125%; EtOH 0.1%; EtOH 0.1%+ DMSO 0.125%) on C2C12 cell morphology

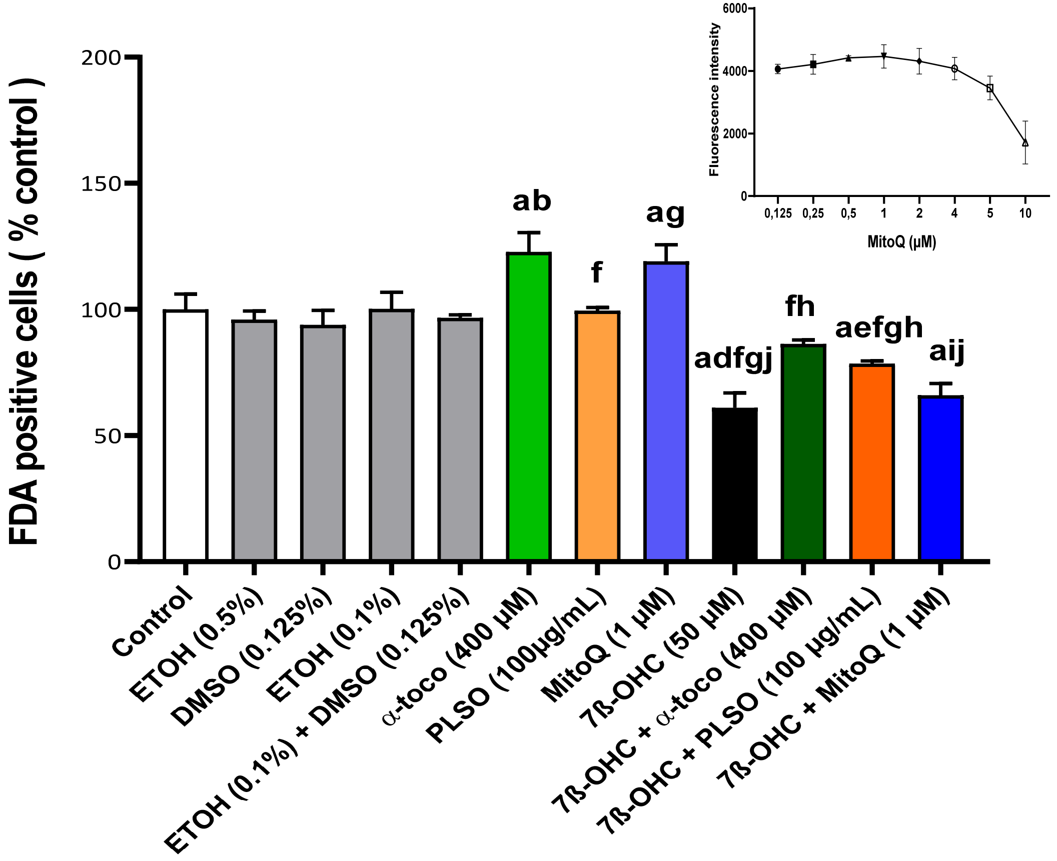

Supplementary Figure S8: Evaluation with the fluorescein diacetate (FDA) assay of the effect of MitoQ on 7β-hydroxycholesterol-induced cell death.
C2C12 cells were incubated for 24 h with or without 7β-OHC (50 µM) in the presence or absence of PLSO (100 µg/mL), *α*-tocopherol (400 µM), or MitoQ (1 µM). Cell viability was then assessed using FDA assay. The concentration of MitoQ (1 µM) was chosen on the basis of a dose effect (see insert). This concentration was in agreement with the MitoQ concentration used on others in vitro studies [69,70]. Data are expressed as percentage of control (untreated cells) and are presented as mean ± standard deviation (SD) from three independent experiments. A multiple comparative analysis between the groups taking into account the interactions was carried out using an ANOVA test followed by a Tukey test. A P-value less than 0.05 was considered statistically significant. The statistically significant differences between the groups, which are indicated by different letters, take into account the vehicle used. **a** : comparison versus control; **b** : comparison versus ETOH (0.5%) ; **c** : comparison versus DMSO (0.125%) ; **d** : comparison versus ETOH (0.1%) ; **e** : comparison versus (ETOH (0.1%) + DMSO (0.125%)) ; **f** : comparison versus *α*-toco (400 µM) ; **g** : comparison versus PLSO (100 µg/mL) ; **h** : comparison versus 7β-OHC (50 µM) ; **i** : comparison versus 7β-OHC (50 µM) + *α*-toco (400 µM); **j** : comparison versus MitoQ (1 µM). No significant differences were observed between untreated (control) and vehicles - treated cells, as well as between 7β-OHC (50 µM) and 7β-OHC+ MitoQ treated cells.

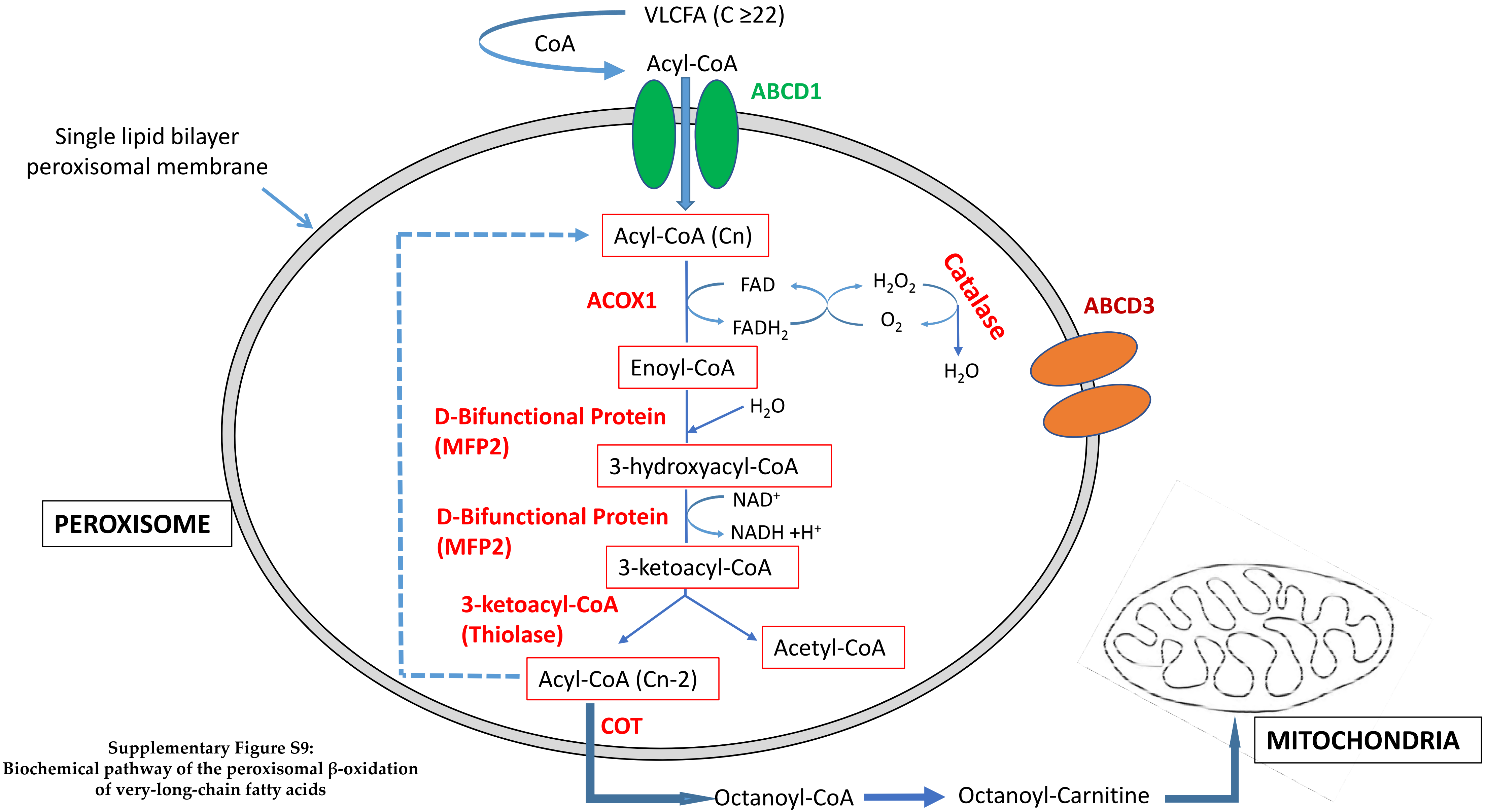

Supplementary Figure S9:
Biochemical pathway of the peroxisomal β-oxidation of very-long-chain fatty acids

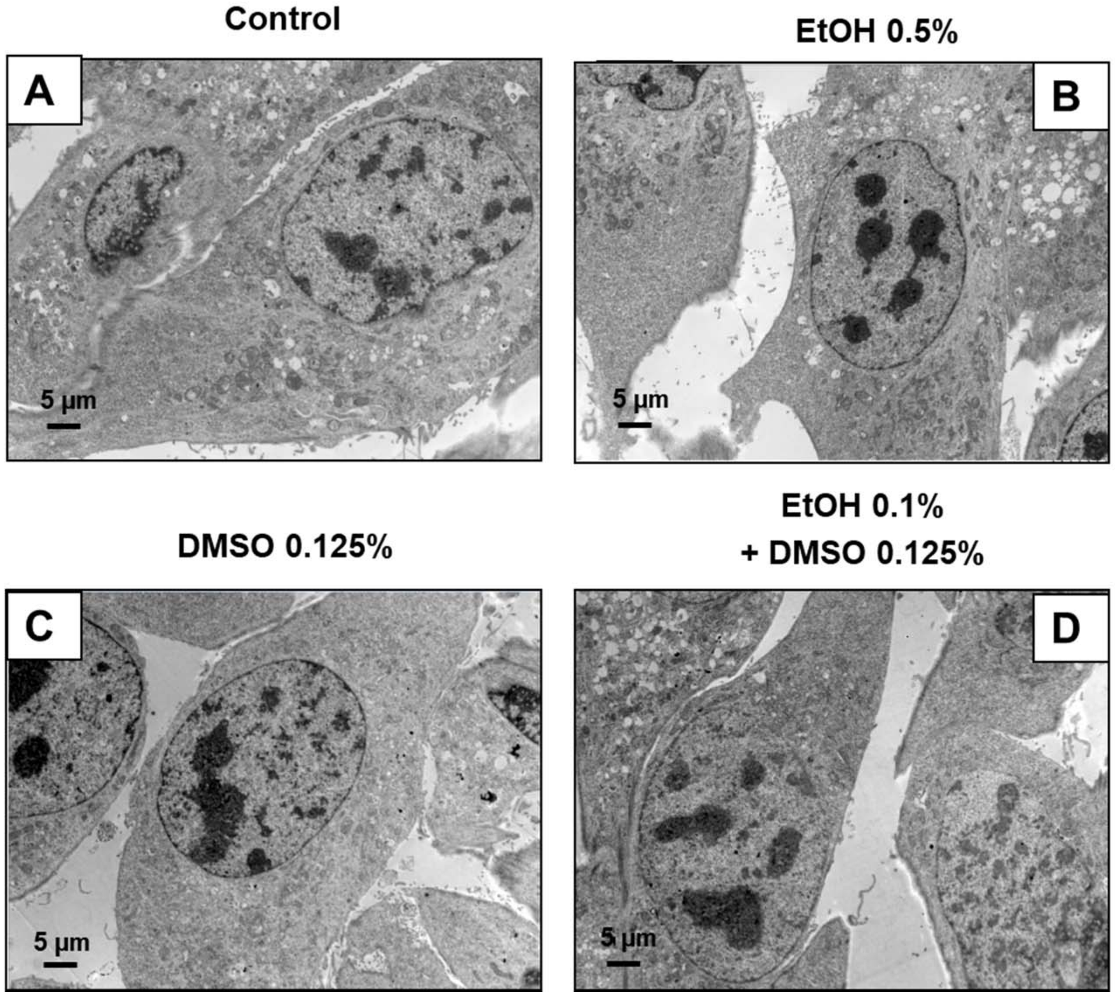

Supplementary Figure S10: Comparison by transmission electron microscopy of cell morphology in control C2C12 myoblasts and vehicles-treated C2C12 myoblasts. In untreated cells (control) **(A)** and vehicles **(**EtOH 0.5% (**B**), DMSO 0.125% (**C**), EtOH 0.1% + DMSO 0.125% (**D**)), C2C12 cells have similar ultrastructural characteristics: they have a fusiform shape, a large central round nucleus with nucleoli, they contain empty cytoplasmic vacuoles and have morphologically normal mitochondria and peroxisomes.

Supplementary Table S1: Available polyphenols spectra present in the database of LARA-Spiral company (Couternon, France)

**A**	Amentoflavone
	Apigenin
**B**	b-resorcylic acid
**C**	Caffeic Acid
	Caftaric acid
	Capsaicin
	Carnosic acid
	Carnosol
	Catechin
	Catechol
	Chicoric acid
	Chlorogenic acid
	Coniferaldehyde
	Coumarin
	c-Resveratrol
	Cryptochlorogenic acid
	Curcumin
	Cyanidin-3-glucoside chloride
**D**	Delphinidin-3-glucoside chloride
	delta-viniferin
	Dihydrocapsaicin
	Diosmin
**E**	Ellagic acid
	Epicatechin
	Epicatechin gallate
	Epigallocatechin
	Epigallocatechin gallate
	epsilon-viniferin
	Eriocitrin
	Eriodyctiol

	Ethylvanillin
	Eupafolin
**F**	Ferulic acid
**G**	Gallic acid
**H**	Hesperetin
	Hesperidin
	Hispidulin
	Homoorientine
	Homovanillic acid
	Hydroxytyrosol
**I**	Isorhamnetin
	Isovitexin
**K**	Kaempferol
	Kaempferol-3-O-glucoside
	Kaempferol-3-rutinoside
	Kojic acid
**L**	Lariciresinol
	Luteolin
**M**	Malvidin-3-glucoside chloride
	Mangiferin
	Matairesinol
	Morine dihydrate
	Myricetin
	Myricetin-3-O-galactoside
	Myricetin-3-O-glucoside
**N**	Naringenin
	Naringenin
	Naringenin-7-glucoside
	Naringin
	Narirutin

	Neochlorogenic acid
	Neohesperidin
	N-vanillynonanamide
**O**	Oleuropein
	Orientin
**P**	p-Coumaric acid
	Peonidine-3-glucoside chloride
	Phloridzin, dihydrate
	p-Hydroxybenzoic acid
	p-Hydroxyphenylacetic acid
	Picateatannol
	Pinoresinol
	Procyanidin A2
	Procyanidin B1
	Procyanidin B2
	Procyanidin B3
	Procyanidin C1
	Protocatechic acid
	Protocatechic aldehyde
**Q**	Quercetin
	Quercetin-3B-glucoside
	Quercetin-3-galactoside
	Quercetin-3-O-b-D-glucuronide
	Quercetin-3-rhamnoside
	Quercetin-4'-glucoside
	Quercetin-3,4'diglucoside
**R**	Resorcinol
	Rosmarinic acid
	Rutin

**S**	Scopoletin
	Secoilariciresinol
	Silibinin
	Silychristine
	Sinapic acid
	Sinapinaldehyde
	Syringaldehyde
	Syringetin
	Syringetin-3-O-galactoside
	Syringetin-3-O-glucoside
	Syringic acid
**T**	Taxifolin
	t-Cinnamic acid
	Teupolioside
	Thymoquinone
	t-piceid
	t-Pterostilbene
	Trans-cinnamaldehyde
	Trans-cinnamyl alcohol
	t-resveratrol
	Tyrosol
**V**	Vanillic acid
	Vanillin
	Verbascoside
	Vitexin-2"-o-rhamnoside
	3,4, 5-trimethoxybenzoic acid
	3,4-Dimethoxybenzoic acid
	3,4-Dimethoxyphenylacetic acid
	2,6-dihydroxybenzoic acid
	6-gingerol
